# Characterization of the G protein-coupled receptor family SREB across fish evolution

**DOI:** 10.1038/s41598-021-91590-9

**Published:** 2021-06-08

**Authors:** Timothy S. Breton, William G. B. Sampson, Benjamin Clifford, Anyssa M. Phaneuf, Ilze Smidt, Tamera True, Andrew R. Wilcox, Taylor Lipscomb, Casey Murray, Matthew A. DiMaggio

**Affiliations:** 1 Division of Natural Sciences, University of Maine at Farmington, Farmington, ME USA; 2 Science Department, Southern Maine Community College, South Portland, ME USA; 3 Department of Biology, Bates College, Lewiston, ME USA; 4 Tropical Aquaculture Laboratory, Program in Fisheries and Aquatic Sciences, School of Forest Resources and Conservation, Institute of Food and Agricultural Sciences, University of Florida, Ruskin, FL USA; 5 Livingston Stone National Fish Hatchery, US Fish and Wildlife Service, Shasta Lake, CA USA

**Keywords:** Molecular evolution, Gene expression, Phylogeny

## Abstract

The SREB (Super-conserved Receptors Expressed in Brain) family of G protein-coupled receptors is highly conserved across vertebrates and consists of three members: SREB1 (orphan receptor GPR27), SREB2 (GPR85), and SREB3 (GPR173). Ligands for these receptors are largely unknown or only recently identified, and functions for all three are still beginning to be understood, including roles in glucose homeostasis, neurogenesis, and hypothalamic control of reproduction. In addition to the brain, all three are expressed in gonads, but relatively few studies have focused on this, especially in non-mammalian models or in an integrated approach across the entire receptor family. The purpose of this study was to more fully characterize *sreb* genes in fish, using comparative genomics and gonadal expression analyses in five diverse ray-finned (Actinopterygii) species across evolution. Several unique characteristics were identified in fish, including: (1) a novel, fourth euteleost-specific gene (*sreb3b* or *gpr173b*) that likely emerged from a copy of *sreb3* in a separate event after the teleost whole genome duplication, (2) *sreb3a* gene loss in Order Cyprinodontiformes, and (3) expression differences between a gar species and teleosts. Overall, gonadal patterns suggested an important role for all *sreb* genes in teleost testicular development, while gar were characterized by greater ovarian expression that may reflect similar roles to mammals. The novel *sreb3b* gene was also characterized by several unique features, including divergent but highly conserved amino acid positions, and elevated brain expression in puffer (*Dichotomyctere nigroviridis*) that more closely matched *sreb2*, not *sreb3a*. These results demonstrate that SREBs may differ among vertebrates in genomic structure and function, and more research is needed to better understand these roles in fish.

## Introduction

G protein-coupled receptors (GPCRs) are cell surface proteins characterized by seven transmembrane domains and the ability to activate G proteins that induce intracellular responses^1^. GPCRs are well-represented across taxa and are highly diverse, with a variety of possible ligands, multiple G proteins, and numerous ways to modulate signaling^2,3^. The importance of GPCRs in physiology, coupled with their accessibility at the cell surface, has attracted considerable research attention in medicine, where they represent some of the most heavily studied drug targets^4^. Despite this attention, functions for some receptors remain poorly understood, and endogenous ligands are unknown (orphan receptors), including many in the Class A Rhodopsin group^5^. One such orphan receptor family is the Super-conserved Receptors Expressed in Brain (SREB)^6–8^. SREBs are found across vertebrates, but not in other chordates or invertebrates, and are characterized by extremely high sequence conservation^8^. These genes have mostly been studied in mammals, where there are three family members: *sreb1* (orphan receptor *gpr27*), *sreb2* (*gpr85*), and *sreb3* (*gpr173*).

The *sreb1* gene exhibits the broadest tissue distribution in mammals^8,9^, and this receptor has been studied in pancreatic β-cells for insulin regulation^10,11^. In mice, *sreb1* knockdown results in decreased insulin mRNA and secretion, as well as glucose tolerance impairment^12,13^. Overall though, SREB1 functions remain largely unknown, as endogenous ligands are still not identified and G protein signals are unclear^13–16^.

The *sreb2* gene is the most conserved member, and also one of the most highly conserved GPCRs throughout vertebrate evolution, exhibiting an identical amino acid sequence between humans and mice^9,17^. Ligands remain unknown for this receptor, but past research has characterized high neuronal expression in the brain that supports a role in neurogenesis^7–9,18, 19^. Changes in *sreb2* expression impact memory and behaviors in mice^17,20^, while variant alleles in humans have been associated with several psychiatric disorders, including schizophrenia and autism spectrum disorder^20–22^.

The *sreb3* gene exhibits elevated expression in the mammalian brain and ovary ^8^, and our understanding of this receptor has greatly expanded with the identification of two possible endogenous ligands: (1) GnRH-(1–5), a cleavage product of the hypothalamic reproductive hormone gonadotropin-releasing hormone (GnRH), and (2) phoenixin (PNX), a cleavage product from small integral membrane protein 20 (SMIM20)^23–25^. GnRH-(1–5)-associated signaling is likely important during brain development for GnRH neuron migration, but the system is complex, as some effects are age-dependent and may use an additional receptor^26–28^. PNX likely acts through SREB3 to regulate diverse processes, including ovarian development and hypothalamic control of reproduction, in addition to other effects in the brain, peripheral nervous system, and heart^29–36^.

Our understanding of SREBs in non-mammalian vertebrates remains limited. However, some conserved roles in fish were recently identified, including SREB1 effects on glucose homeostasis in zebrafish (*Danio rerio*)^37^, and PNX/SREB3 functions in appetite regulation and reproduction in both spotted scat (*Scatophagus argus*) and zebrafish^38–41^. In addition, this system may be critical in regulating zebrafish reproduction in the absence of a functioning hypothalamic GnRH^42,43^. Overall though, many knowledge gaps remain, especially for the *sreb1* and *sreb2* genes, which also exhibit elevated expression in gonads^8,44^ but have rarely been studied in this context. More foundational information on gonadal *sreb* genes in non-mammalian vertebrates is needed to better characterize their reproductive effects. Further, few studies have used an integrative approach across multiple species to broadly study this entire receptor family. To this end, the purpose of the present study was to characterize these genes across fish evolution, using gonadal expression assays and comparative genomics. During these analyses, a novel fourth, fish-specific family member (*sreb3b*) was also identified and characterized. All *sreb* transcripts (*sreb1*, *sreb2*, *sreb3a*, *sreb3b*), as well as a potential ligand (*smim20/pnx*), were assessed in five diverse ray-finned species (Actinopterygii) across evolution.

## Methods

### Study species and animal use approval

Five representative fishes were chosen for gene expression analyses: (1) a holostean fish that diverged before the teleost-specific whole genome duplication event (WGD), the Florida gar (Order Lepisosteiformes, *Lepisosteus platyrhincus*), (2) an ostariophysian teleost, the zebrafish (Order Cypriniformes, *D. rerio*), (3) a teleost with an ovoviviparous (live-bearing) reproductive strategy, the sailfin molly (Order Cyprinodontiformes, *Poecilia latipinna*), (4) a member of the same order but with an oviparous strategy, the African turquoise killifish (*Nothobranchius furzeri*), and (5) a highly-derived perciform, the green-spotted puffer (Order Tetraodontiformes, *Dichotomyctere nigroviridis*). Florida gar, zebrafish, sailfin molly, and green-spotted puffer were maintained and sampled under approved guidelines of the University of Florida (UF) Institutional Animal Care and Use Committee (IACUC #: 202011293). Wild gar were sampled under Florida Fish and Wildlife Conservation Commission permit # FNC-20-002. Zebrafish, sailfin molly, and puffer were maintained at the UF Tropical Aquaculture Laboratory (TAL) (Ruskin, FL, USA). African turquoise killifish samples were provided by the MDI Biological Laboratory (Bar Harbor, ME, USA) under IACUC #: AUP 20-07. All experiments were performed in accordance with the University of Florida and MDI Biological Laboratory IACUC ethical guidelines and regulations for vertebrate animal use. All experiments were conducted in compliance with ARRIVE guidelines. Experimental protocols were approved by University of Maine at Farmington (UMF) Grant Coordination Committee, as well as the University of Florida and MDI Biological Laboratory IACUCs.

### Comparative genomics and puffer *sreb* sequence confirmation

To characterize *sreb* sequences across fish evolution, a comparative genomics dataset was generated using all publicly available fish genomes in Ensembl genome browser version 99 (European Bioinformatics Institute, Hinxton, Cambridge, UK). Briefly, a total of 78 genomes were used, including two agnathans (hagfish *Eptatretus burgeri*, and sea lamprey *Petromyzon marinus*), a member of Class Chondrichthyes (*Callorhinchus milii*), and a lobe-finned fish (Sarcopterygii, coelacanth *Latimeria chalumnae*). The remaining species were all ray-finned fishes (Actinopterygii). For each species, the *D. rerio sreb1* (GenBank Acc. No. NM_001114434.1), *sreb2* (NM_131499.2), and *sreb3* (NM_131498.1) sequences were used to manually BLAST against the genome assembly, and all significant hits (e-value < 1e^−05^) in unique genomic regions were evaluated to identify possible *sreb* orthologous genes. Each gene sequence was exported from Ensembl and used in NCBI-BLAST (Bethesda, MD, USA) against the general nucleotide database to confirm a significant match to the appropriate *sreb* gene. Since some genome assemblies may be incomplete, every gene was also screened through ORFfinder (NCBI) and TMHMM 2.0^45^ to verify a likely full-length amino acid (aa) sequence (~ 370 aa) and confirm the presence of at least most of the canonical transmembrane domains (≥ five out of seven), respectively. Incomplete genes that did not meet these criteria were used only to generate likely gene presence or absence information, and were removed from the dataset.

Following these analyses, the Ensembl 99 *D. nigroviridis sreb1* and *sreb3a* genomic sequences did not meet initial verifications and were labeled as possible pseudogenes with premature stop codons in the genome assembly. To confirm this, puffers (n = 2) maintained at the UF TAL were anesthetized using 100 mg/L buffered Tricaine methanesulfonate (MS-222) and fin clips were preserved in 100% ethanol for DNA analyses at UMF. DNA was extracted using the Qiagen DNeasy Blood and Tissue Kit (Qiagen, Hilden, Germany) and quality checked using a NanoDrop 1000 spectrophotometer (Thermo Fisher Scientific, Wilmington, DE, USA). Primers were designed to flank the protein-coding sequence (Supplementary Table S1) and were used in polymerase chain reactions (PCR) with the Promega GoTaq Flexi PCR kit (Promega, Madison, Wisconsin USA) using standard protocols^46^. PCR products were treated with ExoSAP-IT PCR Product Cleanup Reagent (Affymetrix, Inc., Santa Clara, CA, USA) and transported to the MDI Biological Laboratory for sequencing using the dideoxy chain termination method on an Applied Biosystems 3130xl Genetic Analyzer (Foster City, CA, USA). All PCR products were sequenced in both directions using forward and reverse primers, and sequence chromatograms were trimmed for quality prior to manual assembly. These sequences met full-length verification criteria (ORFfinder and TMHMM). As a result, the *D. nigroviridis sreb1* and *sreb3a* full protein-coding sequences were submitted to GenBank (Supplementary Table S1) and included in the dataset, instead of the original genomic sequences. The complete dataset from the 78 genomes is available as Supplementary Dataset 1. To assess *sreb* sequence conservation, a phylogenetic analysis was conducted. Briefly, a multiple sequence alignment was performed using ClustalW and standard parameters in MEGA X^47^, followed by a maximum likelihood approach in IQ-TREE^48^ with ModelFinder^49^ and 1000 bootstraps using UFBoot2^50^.

### Microsynteny analyses

As the comparative genomics dataset was generated, it became evident that the *sreb3a* gene was consistently absent in any genome from Order Cyprinodontiformes. To distinguish between *sreb3a* gene loss in these species, or a lack of high quality genomic sequencing in these regions, we identified highly conserved flanking genes surrounding the *sreb3a* and *sreb3b* gene locations and performed microsynteny analyses. The *sreb3a* gene locations in nine species that represent a diversity of ray-finned fishes (*L. oculatus*, *D. rerio*, *Ictalurus punctatus*, *Pycocentrus nattereri*, *Esox lucius*, *Amphiprion percula*, *Oreochromis niloticus*, *D. nigroviridis*, and *Gasterosteus oculeatus*) were used to identify the nearest neighboring upstream (*foxp3b*) and downstream (*suv39h1*) genes. Both of these genes in all species were confirmed as significant matches to the *D. rerio foxp3b* and *suv39h1a* genes, respectively, using NCBI-BLAST. The *D. rerio* flanking gene sequences were then compared to all 10 genomes for Order Cyprinodontiformes (*Cyprinodon variegatus*, *Fundulus heteroclitus*, *Gambusia affinis*, *P. formosa*, *P. latipinna*, *P. mexicana*, *P. reticulata*, *Xiphophorus maculatus*, *X. couchianus*, and *Kryptolebias marmoratus*) to identify their presence or absence. A similar procedure was used for analyses involving a novel *sreb* gene cluster (*sreb3b*), except the initial species list across evolution was limited to only those with *sreb3b* (*E. lucius*, *A. percula*, *O. niloticus*, *D. nigroviridis*, and *G. oculeatus*). From this, we identified several highly conserved flanking upstream (*tspy* and *ppp1r3f.*) and downstream (*wdr13*) genes and compared these to the same Cyprinodontiformes genomes. To visualize this, SimpleSynteny ^51^ was used with a selection of the above species, including *L. oculatus*, *D. rerio*, *E. lucius* (an early species to exhibit *sreb3b*), three representative members of Order Cyprinodontiformes (*P. latipinna*, *P. reticulata*, and *K. marmoratus*), and *D. nigroviridis*.

### Amino acid sequence comparisons

To characterize possible functional differences among four putative *sreb* clusters, each gene in the dataset was translated using ORFfinder to obtain likely full-length amino acid sequences (Supplementary Dataset 2). Hagfish (*E. burgeri*), sea lamprey (*P. marinus*), and shark (*C. milii*) genes were not included in these analyses since none of their *sreb* gene sequences clustered in the four major groups. In addition, only SREB sequences with seven transmembrane domains as identified by TMHMM were included. As a result, seven sequences present in Dataset 1 were removed from Dataset 2 before analyses, which included the *Paramormyrops kingsleyae* SREB2A (5 transmembrane domains, TMs), *Denticeps clupeoides* SREB1 (6 TMs), *Salmo salar* SREB3A2 (8 TMs), *Salmo trutta* SREB3A2 (8 TMs), *Hucho hucho* SREB3A1 (8 TMs), *Monopterus albus* SREB2 (6 TMs), and *Lates calcarifer* SREB3B (5 TMs). The final dataset consisted of 75 SREB1, 80 SREB2, 69 SREB3A, and 54 SREB3B sequences across fishes. All sequences were aligned using ClustalW in MUSCLE^52^, and results were visualized using JalView 2^53^. Aligned sequences were parsed manually to identify conservation of canonical functional motifs associated with ligand binding and activation (DRY in TM3, CWxP in TM6, and NPxxY in TM7)^54,55^. Analyses did not include N-terminal regions before TM1, as these were highly variable and some species exhibited multiple potential start sites. Amino acid positions were considered highly conserved if they exhibited three or fewer divergent residues across all species (≥ 96.0%, 96.3%, 95.7%, and 94.4% conservation in SREB1, 2, 3A, and 3B, respectively). In addition, to identify unique characteristics of SREB3B, amino acid positions conserved across SREB1, 2, and 3A but divergent in SREB3B were quantified. To assess if these differences possibly impacted protein structure, I-TASSER^56–58^ was used to predict three dimensional protein models for all SREBs within a selected species that did exhibit SREB3B (*D. nigroviridis*) and one that did not (*D. rerio*). For each prediction, the greatest quality model was assessed based on highest confidence score (C-score ≥ −0.76) and TM-score > 0.5.

### Fish sampling

To characterize gonadal *sreb* gene expression patterns, four species were used that best represented a diversity of ray-finned fishes: gar, zebrafish, sailfin molly, and puffer. Wild adult Florida gar were collected from Orange Lake in Alachua County, FL, USA using standard electrofishing methods and held in live wells until sampling occurred on shore. Gar were then euthanized with an overdose of neutral buffered MS-222 (200 mg/L), and gonads immediately dissected from 12 fish/sex. One gonadal fragment from each fish was preserved in 10% neutral buffered formalin for routine hematoxylin and eosin staining, performed by Histology Tech Services, Inc. (Gainsville, FL, USA), to assess reproductive staging. A second gonadal fragment was immediately preserved in RNALater (Ambion, Inc., Austin, TX, USA), snap frozen in dry ice, and stored at −80 °C until later RNA extractions.

Wild-type adult zebrafish were purchased from a local commercial producer (Segrest Farms, Inc., Gibsonton, FL, USA) and held in a freshwater flow through system at the UF TAL. Zebrafish (n = 12/sex) were euthanized with an overdose of neutral buffered MS-222, and gonads were sampled using similar methods described above, including histological assessments and RNALater preservation.

Adult sailfin mollies were collected from feral populations in outdoor ponds at the UF TAL. Fish (~ 50 individuals) were periodically caught over a two month period using hand nets and baited traps, and euthanized following similar procedures to above. Sampling occurred over a more prolonged time period than the previously discussed species, due to: (1) the ovoviviparious reproductive strategy, and (2) our intent to sample regressed testicular stages. Regarding ovovivipary, female mollies with late stage embryos present within ovaries were common, and a more rigorous sampling scheme was needed to obtain earlier stages. Regarding testicular stages, regressed males were also less common in ponds than those with more developed (spermiating) testes. These additional testicular samples were needed to more fully assess gene expression changes during development, as such stages could not be sampled in either gar or zebrafish, due to difficulties in collecting individuals outside of the spawning season, and small testicular sizes, respectively.

Adult green-spotted puffer were imported through a local wholesaler (Segrest Farms, Inc.) and held in a freshwater flow through system at the UF TAL. Puffers were initially sampled (~ 20 individuals) following above procedures to obtain testicular stages and early ovarian development for histology and RNA extractions. Since this species spawns in saltwater^59^, the remaining puffers were then slowly acclimated to 15 g/L salinity (brackish water conditions) over three months to promote gonadal development. Approximately 20 additional puffers were then sampled to obtain vitellogenic oocytes and more developed testes, which provided a better comparison with previously discussed species that were largely collected in spawning conditions. In addition, to more fully characterize expression of the novel *sreb3b* across other organs, six random puffers (n = 3 individuals/sex) were also sampled for whole brain, eye, gill, heart, liver, and distal intestine.

To compare organ *sreb3b* patterns across multiple fishes, the fifth species, African turquoise killifish (*N. furzeri*), was also included in expression analyses. This species was chosen to compare to puffer patterns because it is a member of Order Cyprinodontiformes and likely lost *sreb* genes in its genome, unlike puffer. In addition, this killifish species is oviparous, which provided a better comparison to puffer than the ovovivparious sailfin molly. Briefly, six adult killifish (n = 3/sex) from the MDI Biological Laboratory Animal Resources Core were removed from a recirculating freshwater system and euthanized with immersion in 1:500 2-phenoxy-ethanol. Killifish were dissected and gonadal stages were identified visually, with all males having developed testes and females in vitellogenic growth. Whole brain, eye, gill, heart, liver, distal intestine, and gonad samples were removed from each fish, preserved in RNALater, and stored at -80 °C until RNA extractions were performed. In addition, to conduct a preliminary study on spatial *sreb3b* expression patterns, a second ovary sample from each female was immediately removed for in situ hybridizations. Ovaries were chosen to enable comparisons between the novel *sreb3b* and prior work on ovarian *sreb3a* in both fish and mammals^35,40^.

### Killifish ovary in situ hybridization

In situ hybridizations were performed following a previously developed protocol^60,61^. Briefly, fresh killifish ovary fragments (n = 3) were frozen on dry ice, sectioned (8 μm) on a cryostat at the MDI Biological Laboratory, and transported to UMF for immediate processing. Custom locked nucleic acid (LNA) probes with 3’ and 5’ DIG labels were purchased from Qiagen (Hilden, Germany) for killifish *sreb3b*, a scramble sequence (negative control), and beta actin (*actb1*, positive control) (Supplementary Table S2). The killifish probe was designed from GenBank Acc. No. XM_015967273 and verified as a member of the fourth *sreb* cluster using NCBI-BLAST. The scramble and *actb1* sequences were designed by Qiagen and verified to not significantly match to any location in the recently published killifish genome or did match to killifish *actb1*, respectively. Probes were diluted according to the miRCURY LNA miRNA Detection Handbook (Qiagen) and hybridized to tissues for 16 h at different temperatures. Anti-DIG antibodies with alkaline phosphatase (Roche Diagnostics, Basel, Switzerland) were incubated for 14 h at room temperature, followed by colorimetric detection for 2.5 h. Slides were mounted using Prolong Gold (Invitrogen, Carlsbad, CA, USA).

### RNA extractions and cDNA synthesis

Preserved gonads from gar, zebrafish, sailfin molly, and puffer were used in RNA extractions, with a subset of individuals in discrete reproductive stages chosen using gonadal histology (n = 4–10 fish/stage, depending on availability). For gar, testis samples were selected with abundant, mature spermatozoa (spermiating, TS, n = 10), along with ovaries with mid-late vitellogenic oocytes (OVV, n = 9). For zebrafish, similar stages and numbers were used (TS and OVV, n = 8 fish/stage). For sailfin molly, more stages were identified, including regressed testes (TR, n = 4), spermiating testes (TS, n = 9), ovaries with neurulation stage embryos (OV + N, n = 8), ovaries with eyed embryos (OV + EE, n = 7), and ovaries with late-stage pigmented embryos (OV + PE, n = 7). For puffer gonads, five stages were also included: regressed testes (TR, n = 5), spermiating testes (TS, n = 9), ovaries with mostly primary growth oocytes (OVP, n = 6), ovaries with early secondary growth (cortical alveoli) oocytes (OVCA, n = 9), and ovaries with mid-late vitellogenic oocytes (OVV, n = 6). For the broad organ surveys in killifish and puffer, all collected samples were used (n = 6 fish/stage, except gonads where n = 3 fish/stage). RNA extractions were performed on all preserved tissues using Tri Reagent (Sigma-Aldrich, St. Louis, MO, USA) and standard phenol/chloroform procedures. In addition, some vitellogenic ovary and liver samples required a third precipitation step using a polyvinylpyrolidone (PVP) solution (2% PVP, 1.4 M NaCl) and 5 M LiCl to remove polysaccharide contamination^62^. Total RNA quantity and quality were assessed using a spectrophotometer and 1.0% agarose gel electrophoresis. Total RNA (2.5 μg for most samples) was treated with ezDNase and a 5 min incubation at 37 °C to remove genomic DNA contamination, following by cDNA synthesis using the Superscript IV VILO kit (Invitrogen).

### Quantitative PCR (qPCR) analyses

To quantify *sreb*-related gene expression patterns, cDNAs were used in species-specific qPCR assays with a StepOne Plus Real Time PCR System and FAST SYBR™ Green Master Mix (Applied Biosystems). Primers were designed using NCBI Primer-BLAST, either from publicly available GenBank sequences or through searching Ensembl genomes for genes with significant similarity to *D. rerio* sequences. Florida gar was an exception, as a genome for this species was not available. Instead, *L. oculatus* sequences were used to search an *L. platyrhincus* Sequence Read Archive (SRA) (SRX3153291) for protein coding fragments, which were confirmed through PCR and sequencing^46^ and submitted to GenBank (Supplementary Table S1). Primers for Florida gar *sreb2* were designed from a previously published sequence (JN853506), and *smim20/pnx* primers were designed from an intact sequence in another SRA (SRX1134593). Regarding killifish assays, no sequences with similarity to *sreb1* were found in the genome or available SRAs (SRP261140), and this gene could not be included. In addition to *sreb*-related genes, three commonly used reference genes (*gapdh*, *eef1a*, and 18S rRNA) were also evaluated in each species (Supplementary Table S3). Primers for *D. rerio eef1a* and *gapdh* were previously designed^63^. Primer sets were verified for intended product amplification using standard PCR and 2.0% agarose gel electrophoresis. All qPCR assays consisted of 10 μl reaction volumes, 1.33 μl diluted template, 0.02–1.0 μM primers (depending on assay), and standard cycling conditions (95 °C for 10 min, 40 cycles of 95 °C for 15 s and 60 °C for 1 min). All samples were assayed in duplicate, and triplicate relative standard curves were made from pooled cDNA. Optimized linear standard curves consisted of four to six points, with approximately 90–100% PCR efficiency and single peak amplification in dissociation curve analysis. Standard qPCR negative controls (no template and no reverse transcriptase) were also used and exhibited no contamination.

For gar, zebrafish, and sailfin molly qPCR assays, results were analyzed using relative quantification^64^ and normalized to 18S rRNA. Other possible reference genes (*gapdh* and *eef1a*) exhibited significant differences among stages (*p* < 0.05) and were not used (Supplementary Fig. S1). In addition, 18S exhibited significant differences among puffer gonad stages (*p* < 0.0001), and these qPCR assays were instead standardized based on an equal quantity of input RNA (2.5 μg/sample) and quantified using the standard curve, similar to previous fish gonadal assays without a reference gene^65,66^. Lastly, organ qPCR surveys in both killifish and puffer also did not exhibit a suitable reference gene (Supplementary Fig. S2). However, these assays could not use a standard input RNA quantity due to small organ sizes (0.6–2.5 μg/sample), and instead assays were normalized based on a modified method^67^, where cell number (cCC) was replaced with input RNA (μg). Other requirements outlined by this method, including similar reagents, volumes, and protocols across samples were followed^67^. To validate this approach, puffer organ assays were also normalized using the Campbell and colleagues method^65^, since most samples (all except heart) consisted of 2.5 μg total input RNA. Similar results in puffer were obtained using both methods. For all assays, results were expressed relative to a testis stage (set to 1.0). Data were expressed as mean ± standard error, and log-transformed relative expression levels were analyzed using two sample *t-*tests or one-way ANOVAs in SYSTAT12 (Systat Software Inc., San Jose, CA, USA). Tukey’s post hoc tests were used to identify significant differences in organ surveys and gonadal stages in sailfin mollies and puffers (*p* < 0.05).

## Results

### Comparative genomics of *sreb* genes

**Figure 1:**
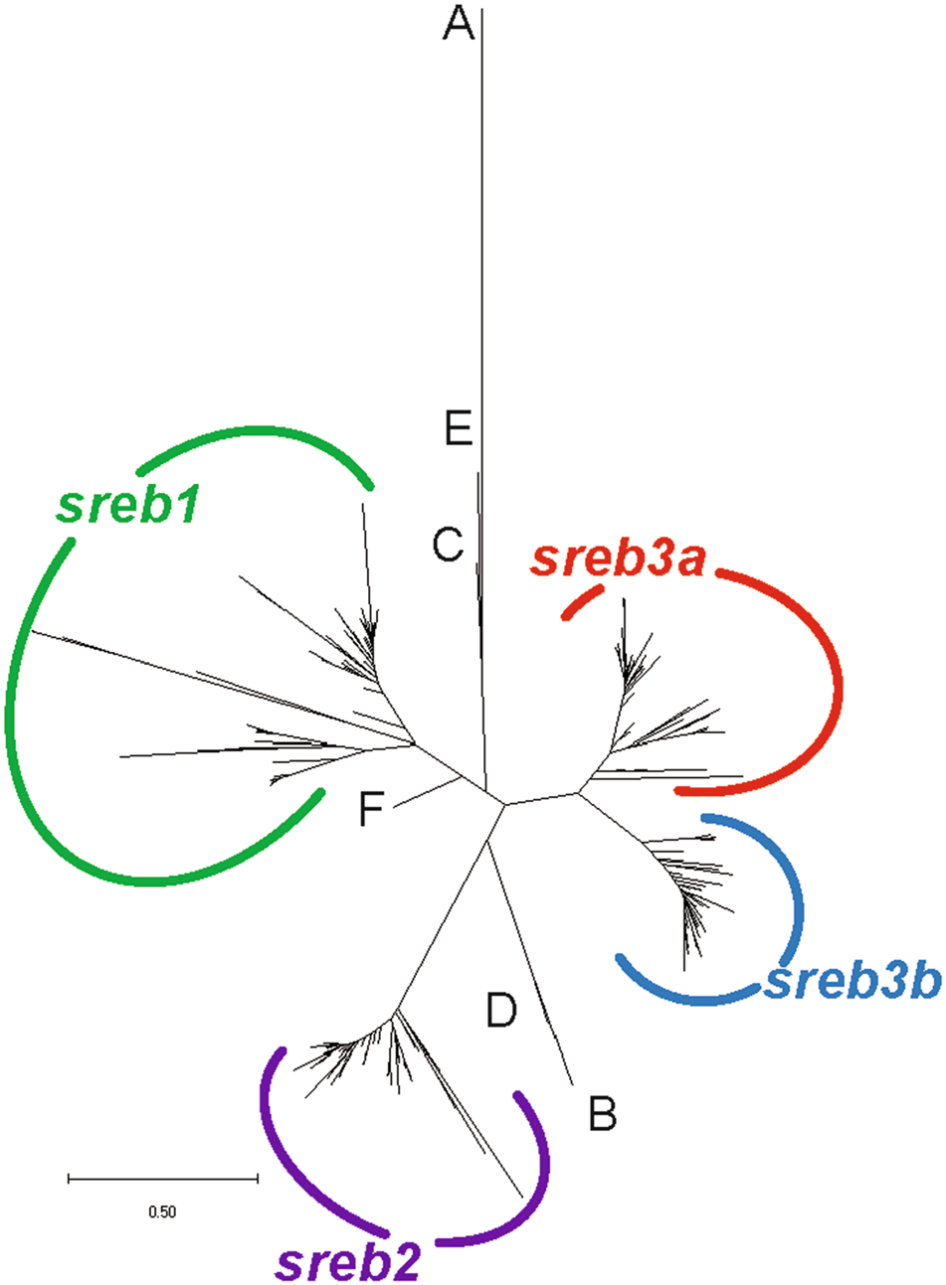
Phylogenetic analysis of all verified fish *sreb* gene sequences available in Ensembl genome browser 99. The tree was generated using a maximum likelihood approach with 1000 bootstraps and rooted to the hagfish *sreb*-like1 gene. See Supplementary Fig. S3 for bootstrap values and species names. Sequences for hagfish (*sreb*-like1 and 2), lamprey (*sreb*-like1, 2, 3) and shark (*sreb*-like1) did not group in four major clusters (labeled **A**–**F**, respectively), while all other genes were identified as *sreb1* (green), *sreb2* (purple), *sreb3a* (red), and a novel *sreb3b* (blue).

**Figure 2:**
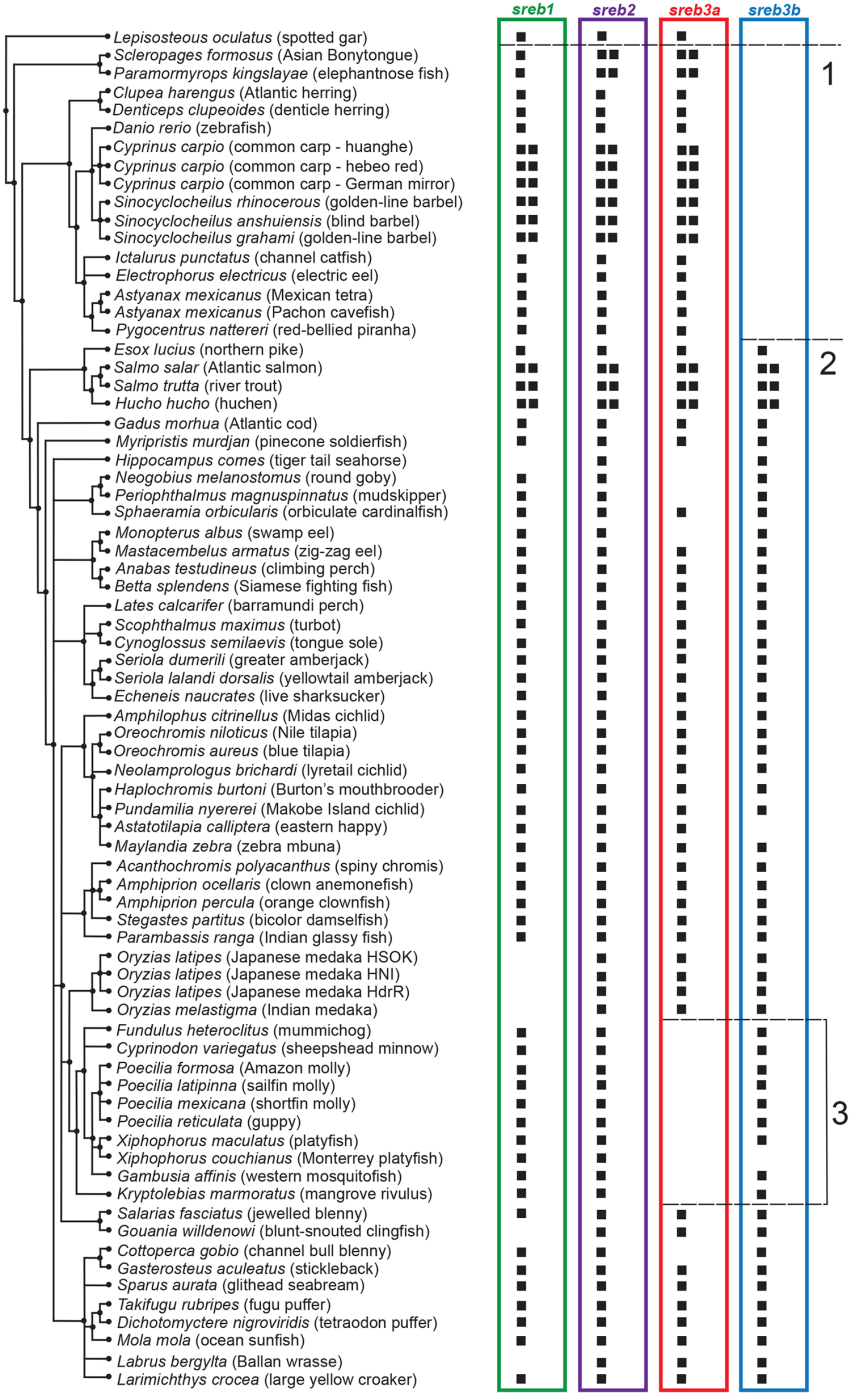
List of all ray-finned fish species (by Ensembl 99 species tree) with presence (black box) or absence (open space) of any unique genomic location with significant similarity to *Danio rerio sreb1*, *sreb2*, or *sreb3a* genes (green, purple, and red, respectively). Sequences with greatest similarity to the novel *sreb3b* were also sorted (blue, see Fig. 1). Dotted lines correspond to: (1) the teleost-specific whole genome duplication event, (2) the first detection of *sreb3b* across fish evolution, and (3) the absence of *sreb3a* in Order Cyprinodontiformes (bracketed region).

**Figure 3:**
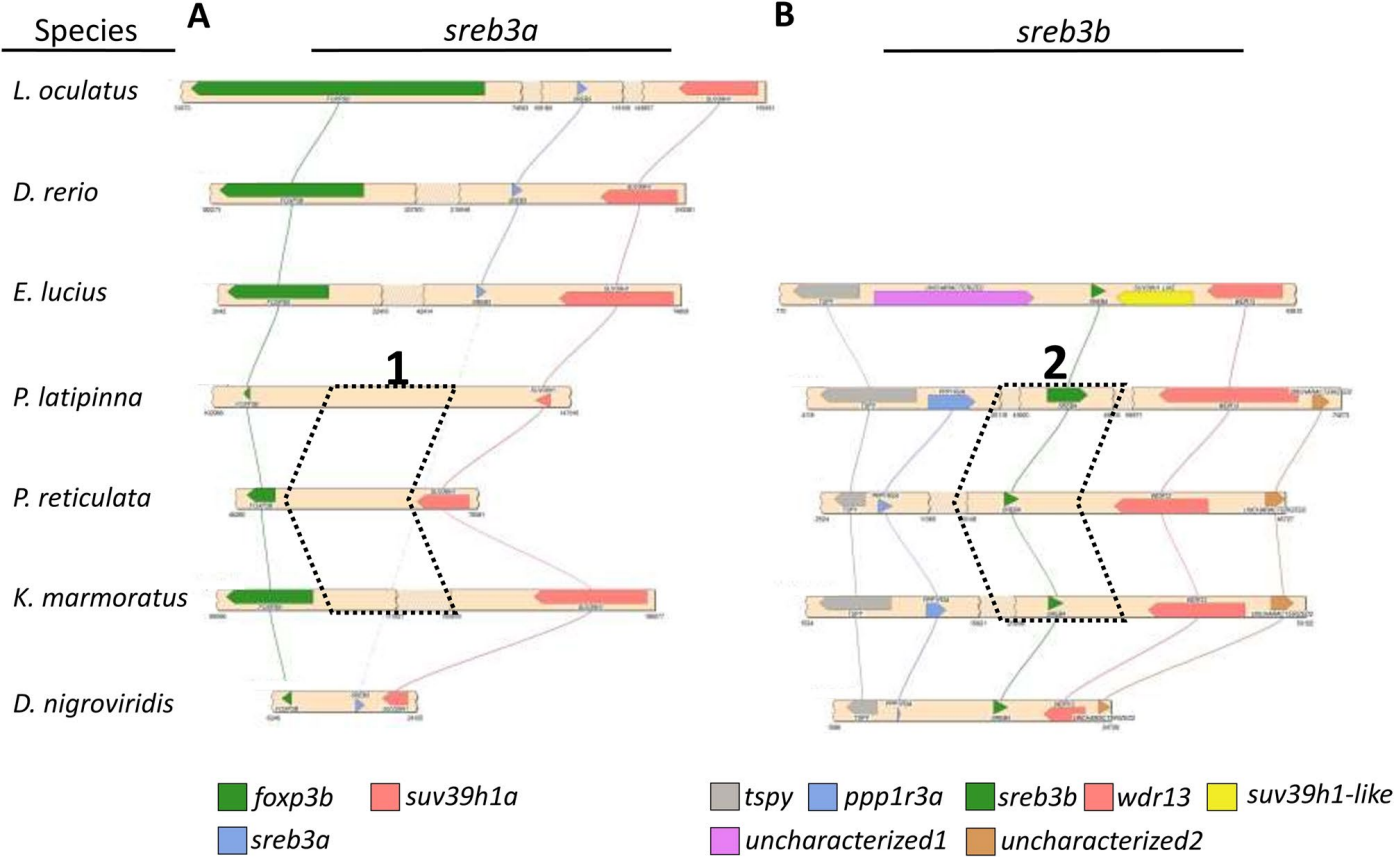
Microsynteny analyses conducted in SimpleSynteny^51^ for seven representative fish species (*Lepisosteus oculatus*, *Danio rerio*, *Esox lucius*, *Poecilia latipinna*, *Poecilia reticulata*, *Kryptolebias marmoratus*, and *Dichotomyctere nigroviridis*) and genomic locations corresponding to (**A**) *sreb3a* and (**B**) *sreb3b*. The dotted blocks labeled 1 and 2 refer to the absence or presence of these *sreb* genes, respectively, in three representative members of Order Cyprinodontiformes. Each bar refers to the species’ genomic sequence, while colored blocks and lines refer to individual genes labeled at the bottom.

Using a phylogenetic analysis, most *sreb* sequences clustered into four groups, except hagfish, lamprey, and shark, which were identified as *sreb*-like genes (Fig. 1). In particular, the hagfish and lamprey genes diverged from each other into two separate groups that could not be clustered (Fig. 1A–E), while the shark *sreb*-like1 gene did exhibit somewhat weak clustering to *sreb1* (Fig. 1F). The fourth cluster, putatively named *sreb3b*, contained sequences from many species labeled in the genome assemblies as *sreb3* but clustered separately from the group that contained the *D. rerio sreb3* sequence (*sreb3a*). The novel cluster exhibited high bootstrap support (100%, Supplementary Fig. S3), although the *sreb3a* cluster was slightly divergent in five species (*L. chalumnae, L. oculatus, Gadus morhua, Salarias fasciatus*, and *D. nigroviridis*). The *sreb3b* gene was not evident in both spotted gar (*L. oculatus*) and early teleost orders (Osteoglossiformes, *Scleropages formosus*; Cypriniformes, *D. rerio*; Siluriformes, *I. punctatus*; and Characiformes, *P. nattereri*), which only exhibited genes that clustered in the first three groups (Fig. 2 line 1). Instead, *sreb3b* was only evident later in euteleosts, with Protacanthopterygii fishes (*E. lucius*, *S. salar*, *S. trutta*, and *Hucho hucho*) being the first evolutionarily to exhibit these genes (Fig. 2 line 2). *Sreb3b* was largely retained in other, more-derived teleosts, even in Order Cyprinodontiformes, where all members may have lost *sreb3a* (Fig. 2 line 3). This was confirmed using synteny, where both the upstream and downstream genes flanking *sreb3a* were present in Order Cyprinodontiformes but no similarity to *sreb3a* itself was detected (Fig. 3A, box 1). However, the *sreb3b* genomic region in most of these species was intact and highly similar to other fishes that exhibited all four genes (Fig. 3B, box 2). The only exception in Order Cyprinodontiformes was the *X. couchianus* genome, which contained sequences with similarity to flanking genes but no identifiable *sreb3b* (data not shown). Later analyses (after Ensembl 99), however, did identify a homologous sequence (XM_028023752) in the appropriate genomic location, and *sreb3b* is likely not lost in this species. In addition, the *E. lucius sreb3b* gene (XM_010890692) was flanked by a *suv39h1*-like gene (Fig. 3B, yellow) with significant similarity and the same orientation to the *D. rerio suv39h1a* gene flanking *sreb3a* (Fig. 3A).

**Figure 4:**
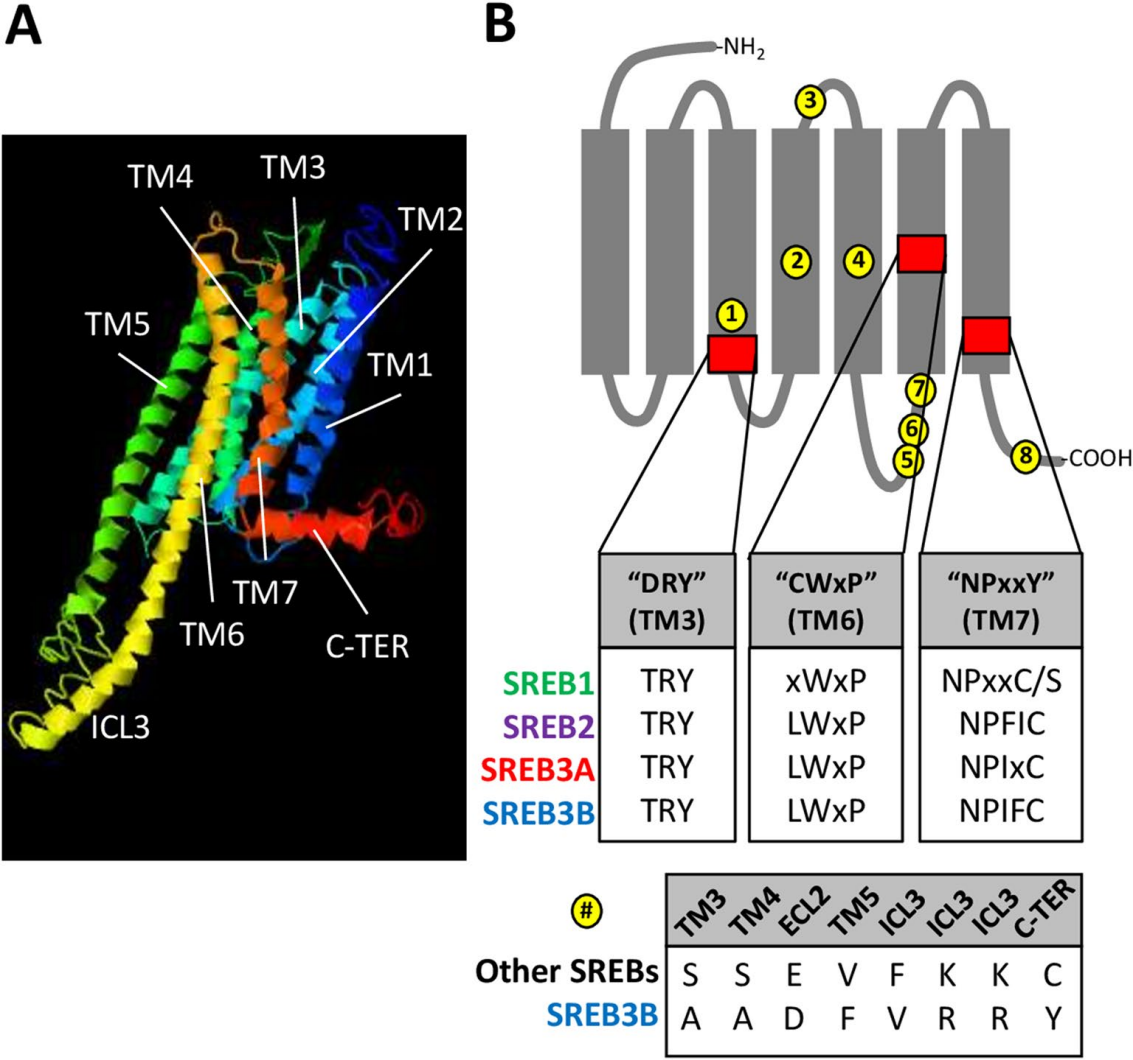
SREB amino acid sequence comparisons. (**A**) Three dimensional model of *Dichotomyctere nigroviridis* SREB1 was made using I-TASSER (C-score = −0.76), to show highly conserved seven transmembrane domains (TM), elongated third intracellular loop (ICL3), and C-terminal region with the eighth α-helix (C-TER). Other models for *D. nigroviridis* SREB2-3B and *Danio rerio* SREB1-3A also exhibited similar characteristics (not shown). (**B**) Identification of highly conserved amino acid positions unique to SREB3B (yellow circles) and SREB deviations from canonical GPCR functional motifs (red squares with first inset). The gray diagram indicates GPCR structure with transmembrane α-helical domains (rectangles, TM1-TM7) and extracellular (top gray lines, ECL) and intracellular (bottom gray lines, ICL) domains. The DRY, CWxP, and NPxxY canonical motifs refer to Nygaard and colleagues^55^. Each amino acid position identified with a letter refers to > 94% conservation across that SREB isoform in the dataset (positions labeled “x” are not highly conserved). The position labeled “C/S” in SREB1 refers to a highly conserved cysteine (C) in many less-derived species but is replaced with serine (S) in more-derived fishes (Neoteleosti). The bottom inset shows the eight residues (yellow) that characterize SREB3B (bottom row) and are divergent but highly conserved across all other isoforms (top row).

To characterize possible functional differences between *sreb3b* and other clusters, amino acid sequences were compared. Overall, SREB protein structure was highly conserved among the four groupings, and all *D. rerio* and *D. nigroviridis* sequences in I-TASSER exhibited canonical G protein-coupled receptor characteristics, including an N-terminal extracellular region, seven α-helical TM domains, an elongated third intracellular loop, and a C-terminal intracellular region with an eighth α-helix (Fig. 4A). However, all SREBs were also characterized by highly conserved differences in three functional motifs associated with G protein-coupled receptor activation (Fig. 4B, red boxes). For instance, the aspartic acid (D) in the TM3 DRY motif was replaced with threonine (T) in most SREB sequences (99.6% conserved across dataset). In addition, the cysteine (C) in the TM6 CWxP motif was replaced with leucine (L) in SREB2-3B (100% conserved) but was more variable in SREB1. The tyrosine (Y) in the TM7 NPxxY motif was replaced with C in SREB2-3B (100% conserved), except in SREB1, where C was found in less-derived fishes but replaced with serine (S) in Neoteleosti. SREB3B was overall similar to other SREBs, except at eight highly conserved amino acid positions that were both unique to this cluster and divergent from SREB1-3A (Fig. 4B, yellow circles). Most of these positions clustered in TM3-5 or intracellular regions. Some of the most likely functional changes included: 1) a shift in TM5 from a smaller valine (V) in SREB1-3A to a residue with a larger hydrophobic side chain (phenylalanine, F) (#4 in Fig, 4B), 2) the opposite shift at a position in the third intracellular loop (#5), and 3) a change in the C-terminal domain from C to a larger, hydrophobic residue (Y) (#8). In order, all eight residues referred to positions 132, 165, 184, 217, 292, 293, 296, and 372, respectively, in the *D. rerio* SREB3A sequence.

### *Sreb* and *smim20/pnx* gonadal expression

**Figure 5:**
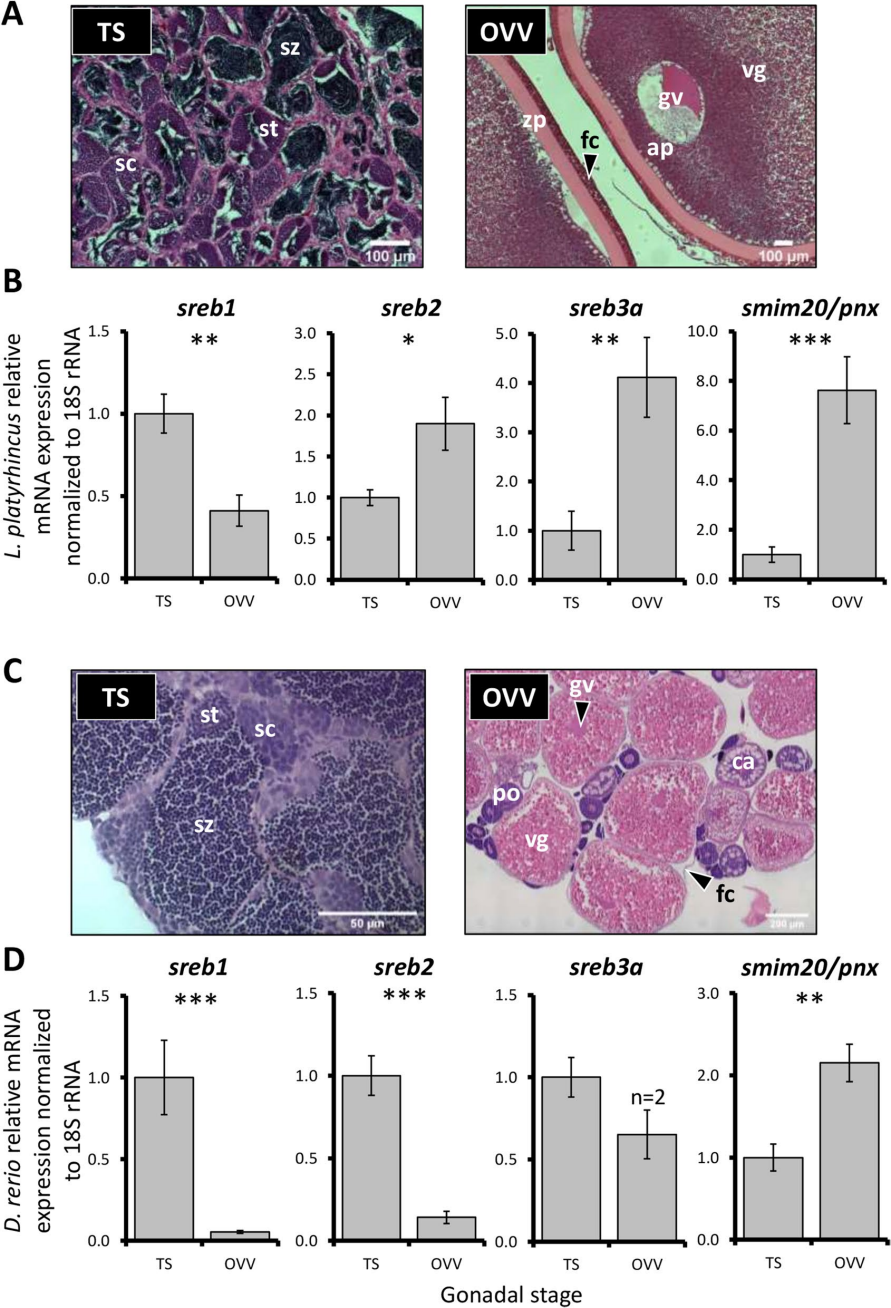
Florida gar (*Lepisosteus platyrhincus*) (**A**) representative gonadal histology and (**B**) relative mRNA expression of *sreb* genes and *smim20/pnx*, normalized to 18S rRNA. Similar zebrafish (*Danio rerio*) stages and relative mRNA expression are shown in C and D, respectively. The TS stage refers to testes with visible spermiation and abundant mature spermatozoa, while OVV refers to ovaries with mid-late stage vitellogenic oocytes. Each bar represents the mean ± standard error, and significant differences are indicated by *(*p* < 0.05), **(*p* < 0.01) or ***(*p* < 0.0001). ap = animal pole, ca = cortical alveoli (secondary) oocyte, fc = follicle cell wall, gv = germinal vesicle, po = primary oocyte, sc = spermatocytes, st = spermatids, sz = spermatozoa, and vg = vitellogenin.

Gar exhibited significant differences in *sreb*-related expression between mature testes and vitellogenic ovaries (Fig. 5A,B). Testes exhibited approximately two fold more *sreb1* (p = 0.004), while other genes (*sreb2*, *sreb3a*, and *smim20/pnx*) were elevated in ovarian tissues by approximately two (*p* = 0.04), four (*p* = 0.001), and eight fold (*p* < 0.0001), respectively. Zebrafish at similar stages, however, exhibited largely opposite patterns, with approximately seven fold more *sreb2* in testes than ovaries (*p* < 0.0001), and *sreb3a* was not detected in most ovarian samples (n = 2, Fig. 5C,D). Zebrafish *sreb1* and *smim20/pnx* were somewhat more similar to gar, with elevated levels in testes (20 fold, *p* < 0.0001) and ovaries (two fold, *p* = 0.002), respectively.

**Figure 6:**
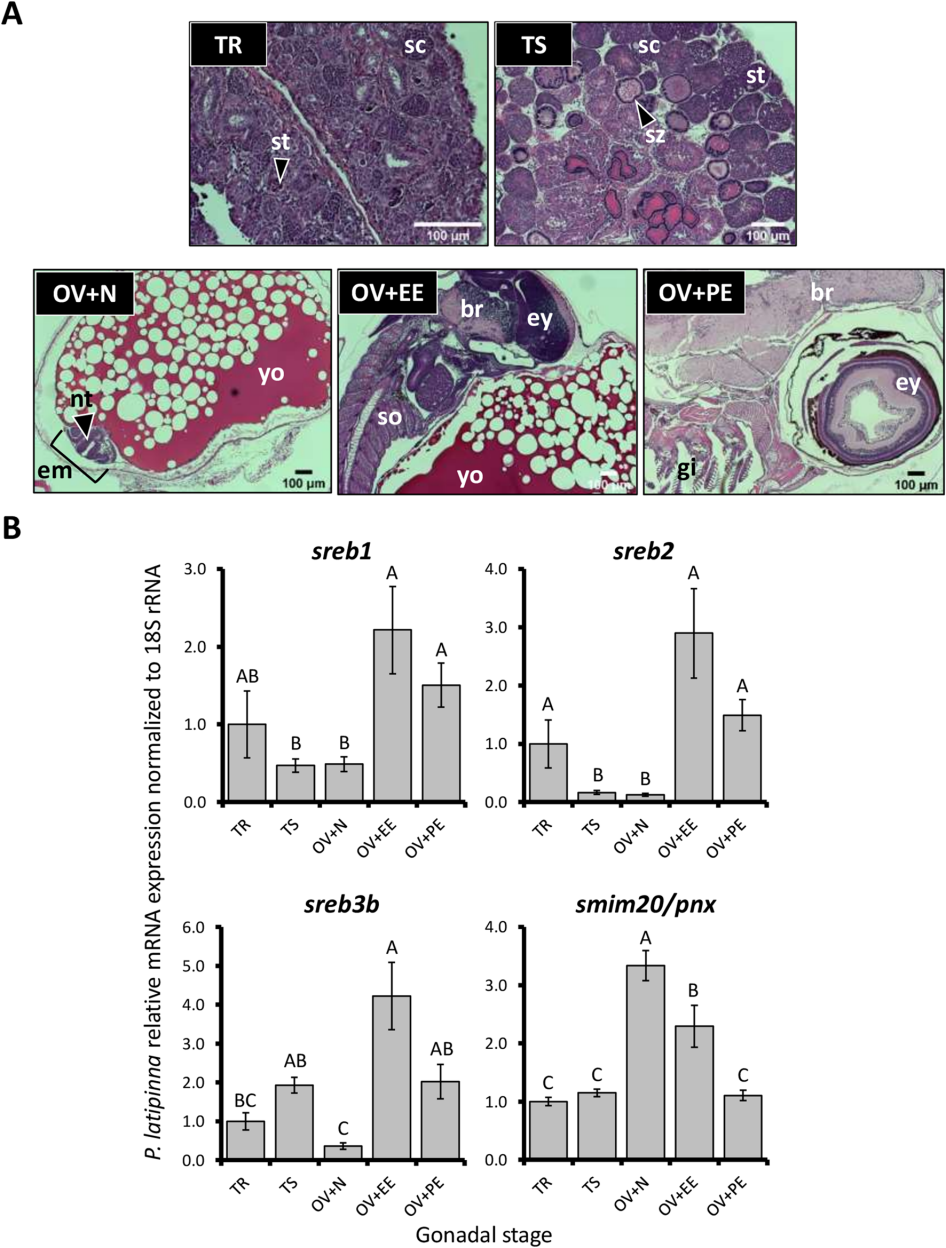
Sailfin molly (*Poecilia latipinna*) (**A**) representative gonadal histology and (**B**) relative mRNA expression of *sreb* genes and *smim20/pnx*, normalized to 18S rRNA. The TR and TS stages refer to regressed testes without spermatozoa and testes with visible spermiation and abundant mature spermatozoa, respectively. The OV + N, OV + EE, and OV + PE stages refer to ovarian tissues with either embryonic neurulation present, eyed embryos, or late-stage pigmented embryos, respectively. Each bar represents the mean ± standard error, and different letters indicate significant differences (*p* < 0.05). br = brain, em = embryo, ey = eye, nt = neural tube, sc = spermatocytes, so = somite, st = spermatids, sz = spermatozoa, and yo = yolk.

Sailfin molly expression patterns were largely dominated by embryonic signals within ovarian tissues, as all *sreb* genes were significantly elevated at the eyed embryo (OV + EE) stage (*p* < 0.0001 for all genes, Fig. 6A,B). Among stages that were more representative of gonadal signals (TR, TS, and OV + N), *sreb2* and *sreb3b* were significantly elevated in testicular tissues, while *sreb1* was largely stable. *Smim20/pnx*, in contrast, was three fold higher at an ovary-dominated stage (OV + N) compared to testes, and expression decreased in later embryonic stages (*p* < 0.0001).

**Figure 7:**
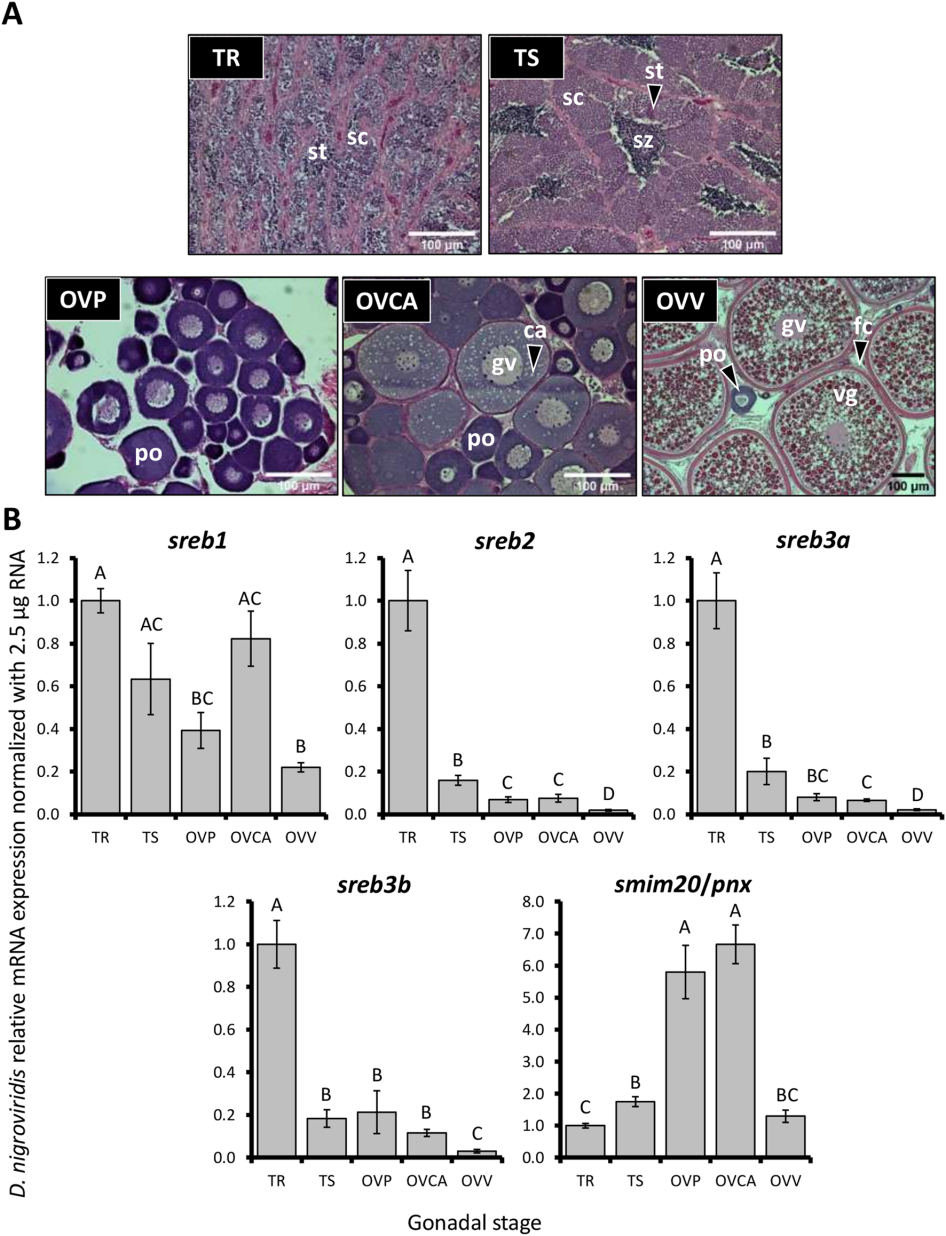
Green-spotted puffer (*Dichotomyctere nigroviridis*) (**A**) representative gonadal histology and (**B**) relative mRNA expression of *sreb* genes and *smim20/pnx*, normalized using stable RNA input across samples (2.5 μg). The TR and TS stages refer to regressed testes without spermatozoa and testes with visible spermiation and abundant mature spermatozoa, respectively. The OVP, OVCA, and OVV stages refer to ovaries with primary oocytes, cortical alveoli (early secondary growth) oocytes, or mid-late stage vitellogenic oocytes respectively. Each bar represents the mean ± standard error, and different letters indicate significant differences (*p* < 0.05). ca = cortical alveoli (secondary) oocyte, fc = follicle cell wall, gv = germinal vesicle, po = primary oocyte, sc = spermatocytes, st = spermatids, sz = spermatozoa, and vg = vitellogenin.

Puffer gonads were overall similar to zebrafish and mollies, with elevated *sreb* levels in testes and greater *smim20/pnx* in ovaries (Fig. 7A,B). *Sreb1* exhibited the most stable expression, with only slightly greater levels in regressed testes compared to primary or vitellogenic stage ovaries (*p* < 0.0001). However, *sreb2*, *sreb3a*, and *sreb3b* patterns were divergent from *sreb1* and highly similar to each other, with approximately ten fold greater expression in regressed testes compared to most ovarian stages (*p* < 0.0001 for all three genes). *Smim20/pnx* exhibited peak expression in early secondary growth and was six fold greater than in testicular stages (*p* < 0.0001), before decreasing during vitellogenesis.

### Characterizing *sreb3b* across organs

**Figure 8:**
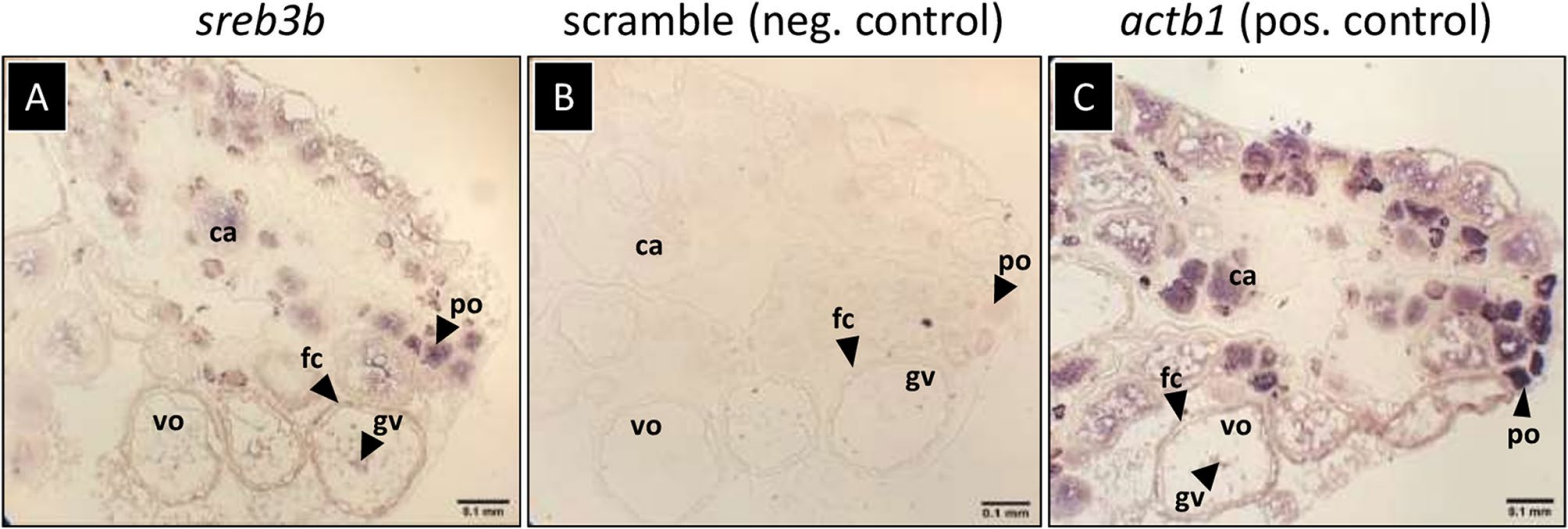
In situ hybridizations of (**A**) *sreb3b*, (**B**) scramble probe (negative control), and (**C**) *actb1* (positive control) in the African turquoise killifish (*Nothobranchius furzeri*) ovary (n = 3). ca = cortical alveoli (secondary) oocyte, fc = follicle cell wall, gv = germinal vesicle, po = primary oocyte, and vo = vitellogenic oocyte.

**Figure 9:**
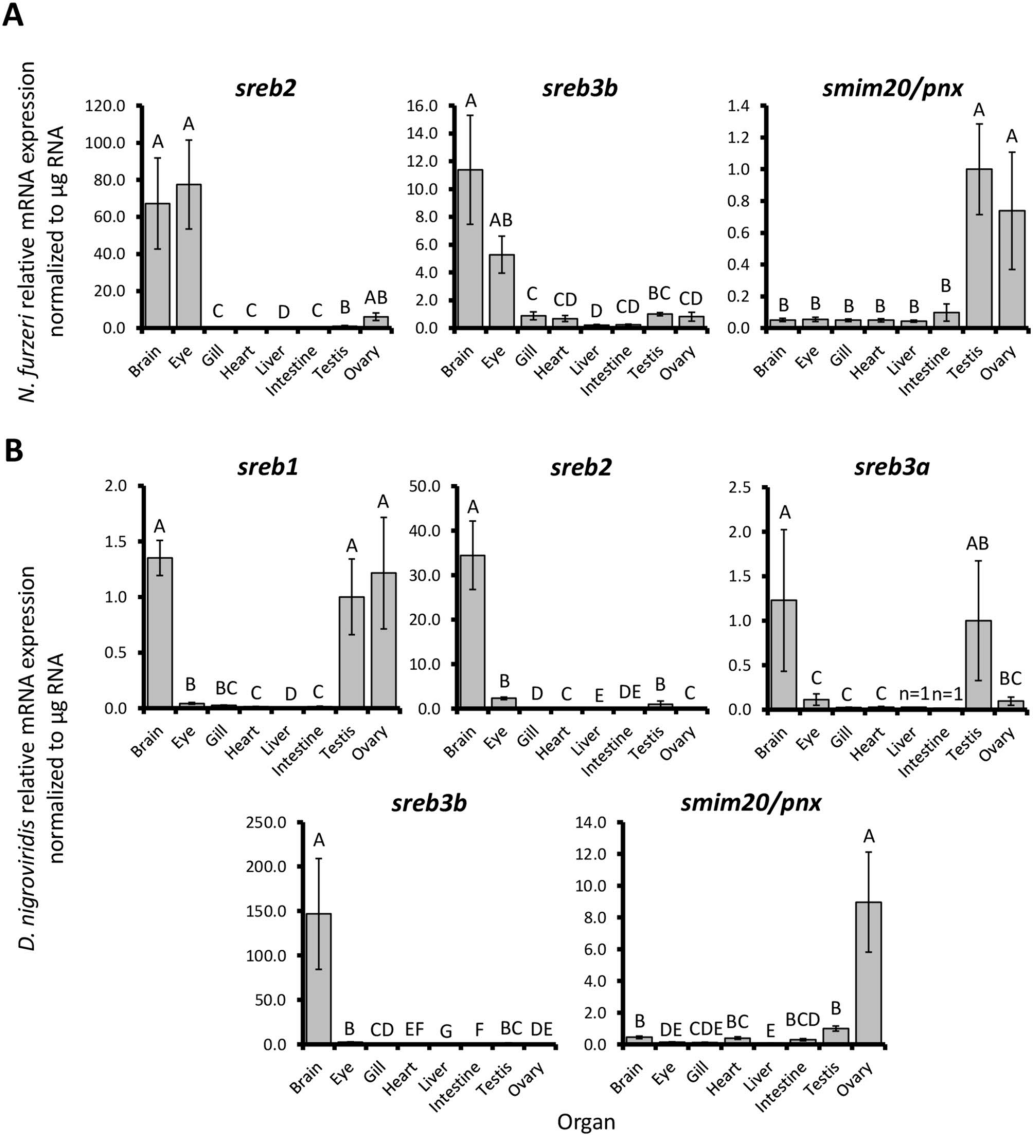
Relative mRNA expression patterns for *sreb* genes and *smim20/pnx* in selected organs of (**A**) African turquoise killifish (*Nothobranchius furzeri*), and (**B**) green-spotted puffer (*Dichotomyctere nigroviridis*). Relative expression levels were normalized to RNA input quantity (0.6–2.5 μg). *D. nigroviridis sreb3a* was not detected in most liver and intestine samples (n = 1 each). Each bar represents the mean ± standard error and different letters indicate significant differences (*p* < 0.05).

To better characterize the novel *sreb3b* gene, expression patterns were assessed spatially within the killifish ovary, as well as broadly quantified with all *sreb*-related genes across both killifish and puffer organs. Killifish ovary *sreb3b* mRNA was detected in all samples both within oocytes and the throughout the somatic follicle cell wall (Fig. 8A). The strongest signals were detected within both primary growth oocytes and follicle cells surrounding vitellogenic oocytes. The negative control scramble probe did not detect any non-specific signal (Fig. 8B), while the positive control *actb1* probe produced abundant signals throughout the ovary (Fig. 8C). Across killifish organs, *sreb2* and *sreb3b* exhibited similar patterns, with elevated expression in brain and eye (*p* < 0.0001 for both genes, Fig. 9A). However, *sreb2* exhibited greater relative expression in the brain than *sreb3b*, compared to testis (67 fold and 11 fold greater, respectively). In contrast, *smim20/pnx* expression in killifish exhibited an opposite pattern, with 20 fold higher expression in gonads than brain. All other organs (gill, heart, liver, and intestine) exhibited largely similar, low overall expression of *sreb*-related genes (Fig. 9A).

Across puffer organs, expression patterns were largely similar to killifish, with some exceptions (*p* < 0.0001 for all genes, Fig. 9B). First, eye signals were overall much lower in puffer and more similar to other organs, as opposed to the brain. Second, *smim20/pnx* patterns were more ovary-dominated and significantly greater than testis. In addition, some gonadal signals in puffer (*sreb1* and *sreb3a*) were highly variable but overall equal to brain levels, which was not detected in killifish. In contrast, *sreb2* and *sreb3b* patterns in puffer were similar and greatly upregulated in the brain (34 and 147 fold higher, respectively, compared to testis), which mirrored patterns in killifish. Overall, these *sreb3b* patterns were more similar to *sreb2* than either *sreb1* or *sreb3a* (Fig. 9B).

## Discussion

The *sreb* genes exhibited high sequence conservation across fishes. Only agnathans and a cartilaginous fish exhibited receptors that could not be easily placed into established groups, which also occurs in other GPCRs^68^. Preliminary synteny analyses in both hagfish and lamprey also confirmed some divergence from more derived-fishes, including one hagfish genomic region that weakly matched flanking genes in both teleost *sreb1* and *sreb2* locations. Based on these patterns and the phylogenetic analysis, *sreb1* and *sreb2* likely emerged before *sreb3* in vertebrate evolution, but the origins of an ancestral vertebrate *sreb* remain unclear. In contrast, other genomic patterns in more-derived fishes were evident, including the teleost-specific WGD^69^. Basal teleosts that diverged early following the event, such as *S. formosus* and *Paramormyrops kingleyae* (Order Osteoglossiformes), exhibited some duplicated *sreb2* and *sreb3a* genes. Additional WGD events in other fishes were also apparent, including carp (Order Cyprinidae), as well as salmonids, but not pike, within the Protacanthopterygii^69,70^. Protacanthopterygii fishes were also the first lineage to exhibit evidence of a novel fourth member, putatively named *sreb3b* or *gpr173b*, which was found as a single gene in northern pike (*E. lucius*). This evolutionary placement indicates that *sreb3b* likely did not emerge from the teleost-specific WGD, but instead arose later in the Euteleosti lineage as a separate gene duplication event from *sreb3*. The exact origin of *sreb3b* within the euteleosts is difficult to identify without greater resolution though, as no genome was available for the closely related Order Lepidogalaxiiformes or other groups within the Protacanthopterygii^71^. The *sreb3* duplication event hypothesis, however, is also supported by the presence of flanking *suv39h1* genes in both *E. lucius* genomic regions. Following duplication, this *suv39h1*-like gene was likely lost in more-derived teleosts, while the *sreb3b* gene diverged and was retained.

The *sreb3b* gene is overall characterized by high similarity to other family members. For instance, all fish SREBs exhibited similar protein structures and GPCR functional motifs, which are also shared in the three mammalian receptors but differed from canonical sequences^8,55^. Although functional implications for these changes are unknown in SREBs, the interaction between arginine (R) and asparagine (D) in the DRY motif is generally important in keeping an inactive conformation^55^, and interactions with NPxxY in TM7 are important for receptor activation and G protein signaling^72^. The highly conserved amino acid changes in both motifs may therefore limit the inactive state and contribute to constitutive activity previously identified for some SREBs^14^. Roles for other conserved amino acid changes, including the first position of the CWxP motif and the specific Neoteleosti SREB1 change to S in NPxxY, are unknown and require more investigation. However, none of these motifs were divergent in SREB3B. Instead, the novel receptor was characterized by eight unique and highly conserved amino acid changes in other regions. These changes were clustered around TM3-5 and the third intracellular loop, which are known to be important in ligand binding and G protein signaling, respectively^55^. SREB3B may differ from SREB3A in its ability to bind the PNX or GnRH-(1–5) ligands, or the receptor may use alternative intracellular pathways.

*Sreb3b* expression patterns were also similar to other family members. Spatial patterns within the ovary suggest both oocyte-derived and follicle cell expression, which largely mirrored *sreb1* and *sreb3a* in zebrafish, Atlantic cod, and human ovary^35,40,44,66^. *Sreb3b* expression within follicle cells may function similarly to *sreb3a*, which regulates cell proliferation and follicular progression through vitellogenesis and maturation^35,40^. In contrast, *sreb*-related oocyte expression may be important as stored maternal transcripts for early embryonic development. In the organ surveys, some differences were detected between *sreb3b* and other receptors. Here, *sreb3b* exhibited the highest expression profile in the puffer brain. Puffer *sreb3b* patterns most closely matched *sreb2*, not *sreb3a*, and these characteristics were similar in killifish. As such, retention and divergence of *sreb3b* following its gene duplication event may be associated with brain-related functions. This high similarity between *sreb2* and *sreb3b* is also evident across genomes, where both were identified in nearly all possible genome assemblies, while the other receptors (*sreb1* and *sreb3a*) have likely been lost in some lineages.

The functional significance of *sreb3a* gene loss in Order Cyprinodontiformes is unknown, but it does highlight that *sreb* sequence conservation across vertebrates does not indicate identical functions in all species. Indeed, *sreb3a* loss may not even be unique to fish, as preliminary data by our group also suggest that this gene may be absent across birds. Although *sreb3* loss has been confirmed in chicken^73^, this is difficult to verify in other species due to a lack of conservation in these genomic regions. Similarly, *sreb1* loss in both killifish and medaka (genus *Oryzias*, Fig. 2) is not certain, but searches in transcriptomic databases and across genome assemblies in multiple species have not yet identified an orthologous sequence. In the future, these differences may be useful to further our understanding of *sreb*-related gene functions, such as investigations of hypothalamic PNX across diverse fishes.

Using a comparative approach, it was also evident that gonadal *sreb* patterns are not similar across fish evolution, while *smim20/pnx* is likely highly conserved. For instance, gar receptors were ovary-dominated for *sreb3a* and more stable for *sreb1*, which may reflect involvement in similar processes to mammals. Teleosts, however, were instead characterized by elevated testicular expression for most receptors, regardless of reproductive strategy. SREB roles in testes are poorly understood, but testicular *sreb3a* was detected in zebrafish somatic support cells, and PNX induces upregulation of steroidogenesis-related expression and *dmrt1*, which is critical in male development^40,74^. As such, elevated *sreb* during early testicular stages in both sailfin molly and puffer likely reflect effects in promoting testicular development. In contrast, *smim20/pnx* patterns were highly conserved across fishes, with elevated patterns in ovaries that were particularly high in early stages. This pattern is characteristic of an oocyte-derived message that may be stored for embryonic development^44^. Indeed, both *sreb* and *smim20/pnx* patterns were elevated in sailfin molly embryos, which likely suggests their importance in nervous system development across vertebrates^20^. However, since PNX is a post-translational cleavage product, the *pnx* mRNA signal is linked to mitochondrial *smim20*, and expression patterns here may better reflect broader SMIM20 roles in respiratory chain assembly^25^ as opposed to PNX-specific functions. As such, caution is necessary when interpreting *smim20/pnx* patterns. Overall though, these patterns suggest that SREBs may play an important role in teleost early testicular development, and more work is needed to better understand these functions.

## Conclusion

Although the SREB family is highly conserved across vertebrates, some fish exhibit characteristics that diverge from mammals, including *sreb3a* gene loss and the acquisition of a novel *sreb3b* gene. *Sreb3b* did not arise during the teleost WGD, but instead likely originated later as a gene duplication event of *sreb3* in euteleosts. This novel receptor exhibits expression patterns similar to other family members but most closely matches patterns for *sreb2*, not *sreb3a*. Gonad-specific patterns across teleosts were largely similar for all *sreb*-related genes and suggests important roles in testicular development, while gar exhibited patterns somewhat closer to mammals. Further research is needed to identify unique roles for *sreb3b* in specific brain regions, and gonadal functions for all *sreb* genes should be evaluated. To this end, future SREB research could benefit from an integrated approach across multiple family members, and recent work in the fish PNX/SREB3 system will be invaluable as functions become better understood.

## Supplementary Information

Supplementary Information 1.

Supplementary Information 2.

Supplementary Information 3.

## Data Availability

All data generated or analyzed during this study are included in this published article and its Supplementary Information files.
