# Supplementary material for: Characterization of the G protein-coupled receptor family SREB across fish evolution
Source: Sci Rep. 2021 Jun 8;11:12066. doi: 10.1038/s41598-021-91590-9 (PMC8187511; doi:10.1038/s41598-021-91590-9)
Supplement: Supplementary file 3 — Supplementary Information 3. [file 41598_2021_91590_MOESM3_ESM.pdf]

Supplementary Information for:

Characterization of the G protein-coupled receptor family SREB across fish evolution

Timothy S. Breton[1*], William G.B. Sampson[1], Benjamin Clifford[2], Anyssa M. Phaneuf[1], Ilze Smidt[3], Tamera True[1], Andrew R. Wilcox[1], Taylor Lipscomb[4, 5], Casey Murray[4], Matthew A. DiMaggio[4]

[1]Division of Natural Sciences, University of Maine at Farmington, Farmington, ME, USA

[2]Science Department, Southern Maine Community College, South Portland, ME, USA

[3]Department of Biology, Bates College, Lewiston, ME, USA

[4]Tropical Aquaculture Laboratory, Program in Fisheries and Aquatic Sciences, School of Forest Resources and Conservation, Institute of Food and Agricultural Sciences, University of Florida, Ruskin, FL, USA

[5]Livingston Stone National Fish Hatchery, US Fish and Wildlife Service, Shasta Lake, CA, USA

*Corresponding author: timothy.breton@maine.edu

Table S1. Gene symbols, primer sequences, product sizes (bp), and GenBank accession numbers for coding domain sequence (cds) fragment (*Lepisosteus platyrhincus*) or complete cds (*Dichotomyctere nigroviridis*) sequences. The *L. platyrhincus sreb2* and *smim20/pnx* fragments were previously available as a partial cds sequence (JN853506) and sequence read archive fragment (SRX1134593), respectively, and were not sequenced in this study. Only *D. nigroviridis sreb1* and *sreb3a* were sequenced due to being identified as possible pseudogenes in Ensembl version 99. GenBank accession numbers for *D. nigroviridis* sequences refer to manually assembled complete cds sequences from both primer sets. Set 1 and set 2 primers amplifed approximately the first and second half of the cds, respectively.

Species	Gene	Primer sequence (5'-3')	bp	GenBank
L. platyrhincus	*sreb1*	F - AGCAACACTTCGCTCCAGAA<br>R - TGTGGTTCCCACGATGACAG	573	MN937260
	sreb3a	F - CGATGTGGGCACCTACAAGT<br>R - ACATGACGCAGTAGGGTTCG	641	MN937261
	eef1a	F - CAAGGAAGTCAGCGCCTACA<br>R - TCTCCACACACATGGGCTTC	728	MN937262
	gapdh	F - GCCAACATCAAGTGGGGAGA<br>R - CACTCTGGTGCTGTAGCCAA	726	MN937263
	18S rRNA	F - GAGTCGGCATCGTTTATGGT<br>R - CCGCAGCTAGGAATACTGGA	247	MN944469
D. nigroviridis	*sreb1*<br>(set 1)	F - TTCGGAGGGGGTTTTGTCTG<br>R - TCCACACCATGCAGATGACC	673	MN937264
	sreb1<br>(set 2)	F - GGTCATCTGCATGGTGTGGA<br>R - AAAGGTGAGGTGGAGGAGGT	720	---
	sreb3a<br>(set 1)	F - TCAGGTGACCATGTGGAAGC<br>R - AGCGTGTCGTTAGCCTTGAA	812	MN937265
	sreb3a<br>(set 2)	F - TTCAAGGCTAACGACACGCT<br>R - TGGTTCCCTTGCTGTTGTGT	686	---

Table S2. *In situ* hybridization locked nucleic acid (LNA) probes used for *Nothobranchius furzeri sreb3b*, a negative control scramble sequence, and positive control beta actin (*actb1*). Scramble and actb1 sequences were designed by Qiagen Corporation, while the *sreb3b* sequence was designed from GenBank Acc. No. XM_015967273.1. Tm refers to the hybridization temperature used. Scramble refers to a probe sequence with no significant similarity to any sequence in the *N. furzeri* genome. All probes were 3' and 5' DIG labeled for procedures.

Gene	Tm	Sequence
sreb3b	54	TACAAGGCTGACACAGATGAT
Scramble	57	GTGTAACACGTCTATACGCCCA
actb1	57	CTCATTGTAGAAGGTGTGGTGCCA

Table S3. qPCR primer sequences, product sizes (bp), PCR efficiencies (%), and Mean Ct values for genes studied in five fishes (*Lepiosteus platyrhincus*, *Danio rerio*, *Poecilia latipinna*, *Nothobranchius furzeri*, and *Dichotomyctere nigroviridis*). Mean Ct refers to the 1/40 standard curve point in each assay. In *D. nigroviridis*, PCR efficiencies and Mean Ct before and after slashes refer to values for the organ distribution and gonad-specific assays, respectively.

Species	Gene	Primer sequence (5'-3')	bp	PCR efficiency (%)	Mean Ct
L. platyrhincus	*sreb1*	F - CTCTGGACTTGTGTGGCTGT<br>R - TGCACTGCTCTTCCTCCTTG	115	107.6	30.54
	sreb2	F - TCTGGCTGTCATCTGCATGG<br>R - TCATTGGCTCGGAAGGATCG	135	91.4	28.67
	sreb3a	F - CACCATACCTCACCGCTACC<br>R - GGAGGAAGCAGATGATGGGG	83	93.7	29.13
	smim20/pnx	F - CCAGTTCTGTTCACTTGCTGA<br>R - TGCTGTCGGTGTTGCATTTTA	90	98.6	20.45
	eef1a	F - CAAGGAAGTCAGCGCCTACA<br>R - CCCCTTAAACCAGGGCATGT	127	89.2	20.85
	gapdh	F - CCCAATGTCTCCGTGGTTGA<br>R - CTTCATGGGTCCCTCAGCAG	102	103.2	18.95
	18S rRNA	F - AGTCGGCATCGTTTATGGTC<br>R - CGAAAGCATTTGCCAAGAAT	102	90.2	9.68

Table S3, cont.

D. rerio	*sreb1*	F - CTGGCGTCTTTGGGTCTCAT<br>R - TGGAGCGAGCTGTCTTTCAG	86	105.6	34.81
	sreb2	F - CTTCCTGCTGGACCTCTGTG<br>R - GTCCAGGCTGAGCCATTCTT	93	107.3	29.48
	sreb3a	F - TGAGCTTTGCTCAAGCAGGA<br>R - AGTAGAGGAGCGTGGTGCTA	111	99.5	26.52
	smim20/pnx	F - TGAAGACTACAAGCAAGTGCAGA<br>R - AGGGATCAGACCAGACCTTCA	92	100.5	22.83
	eef1a	F - CTTCTCAGGCTGACTGTGC<br>R - CCGCTAGCATTACCCTCC	358	107.6	13.33
	gapdh	F - GTGGAGTCTACTGGTGTCTTC<br>R - GTGCAGGAGGCATTGCTTACA	173	98.9	13.48
	18S rRNA	F - AAACGGCTACCACATCCAAG<br>R - TTACAGGGCCTCGAAAGAGA	109	92.0	8.06
P. latipinna	*sreb1*	F - TTCGAACACCGCTACGTCAA<br>R - GGTCGTAAACGAAGCACAGC	115	106.2	25.60
	sreb2	F - TCGGTGTGCTCTCCTGTTTC<br>R - AGAACGTCAGCCTCTTGGTG	108	99.3	27.62
	sreb3b	F - ACCCTGCTATTCCTCGTCCT<br>R - GAAGCTCATCCACACCGTGA	117	106.3	24.93
	smim20/pnx	F - TCTGGTCCGATCCGTTCAAG<br>R - CGCATCCAAAGCCGTCAAAA	149	94.8	21.25
	eef1a	F - GGCATCTCCAAGAACGGACA<br>R - AGCGCTTCTGGCTGTAGTTT	124	106.5	27.77
	gapdh	F - CCGTTCGTCTGGAGAAACCA<br>R - GCCGTTGAAGTCTGTGGAGA	128	105.9	16.96
	18S rRNA	F - CCCGAGATCCAACTACGAGC<br>R - ATTGGAGGGCAAGTCTGGTG	99	87.1	9.87

Table S3, cont.

N. furzeri	*sreb2*	F - TTTGTTGGACCTGTGCACCT<br>R - CACCCAGGAAGGCAATCACT	131	92.4	28.96
	sreb3b	F - CATGCTGGTGCTGAGAGACA<br>R - CACTGTAAGTCCAGGCCGAG	146	101.1	29.75
	smim20/pnx	F - ATTCGGAGGCTTCGTTACGG<br>R - CTGGTTAATTCCTGCCCGGT	118	109.1	20.85
	eef1a	F - CTTCGTCCCCATTTCTGGCT<br>R - CGGTTGATCTTCCAGCCCTT	87	89.7	26.58
	gapdh	F - CTGTCGGCAAGGTCATTCCT<br>R - CGGTCAGATCCACCACTGAC	93	92.9	20.41
	18S rRNA	F - GGACCCTCGAACCTCCTAGT<br>R - GCTGACGGAGGAAAGGAGAG	88	100.1	17.03
D. nigroviridis	*sreb1*	F - CTTCCTGCTCTTCTGCGTCA<br>R - CGCACGTACACACGTTCATC	89	103.7 / 99.7	29.59 / 28.01
	sreb2	F - ACTCCTTGGGCTTCATGCTC<br>R - GGCTAACAGCAGGCACAAAC	129	91.4 / 103.3	29.47 / 28.95
	sreb3a	F - GCACGTACAAGTTCATCCGC<br>R - GCATTAGCATGAAGCCCAGC	90	90.9 / 97.5	31.89 / 32.39
	sreb3b	F - TCACGGTGTGGATGAGCTTC<br>R - GCAGCACTTTCCTCAGGTCT	81	104.2 / 100.0	27.33 / 29.03
	smim20/pnx	F - GACGGAATCAACCAGGCAGA<br>R - GGAATCATTTTCCGGCAGGC	82	113.7 / 102.1	20.20 / 19.40
	eef1a	F - TGAGGCCGGTATCTCCAAGA<br>R - AAACGTGCCTGACTGTAGGG	132	97.8 / 93.5	14.53 / 11.85
	gapdh	F - CTGTCCAAGCCTGCGTCTTA<br>R - ATGGAGGAGTGAGAGTCCCC	137	102.9 / 97.9	16.96 / 22.88
	18S rRNA	F - CGCTACTACCGATTGGATGG<br>R - GTAATGATCCTTCCGCAGGT	163	93.0 / 94.2	13.07 / 14.73

Fig. S1. Relative mRNA expression for putative qPCR reference genes that did not exhibit stability across gonadal reproductive stages in A) Florida gar (*Lepisosteus platyrhincus*), B) zebrafish (*Danio rerio*), C) sailfin molly (*Poecilia latipinna*), and D) green-spotted puffer (*Dichotomyctere nigroviridis*). Each assay was normalized to either 18S rRNA or input RNA quantity, depending on species. Each bar represents the mean ± standard error, and significant differences are indicated by * ($p < 0.05$), ** ($p < 0.01$) or *** ($p < 0.0001$), or different letters indicate significant differences ($p < 0.05$).

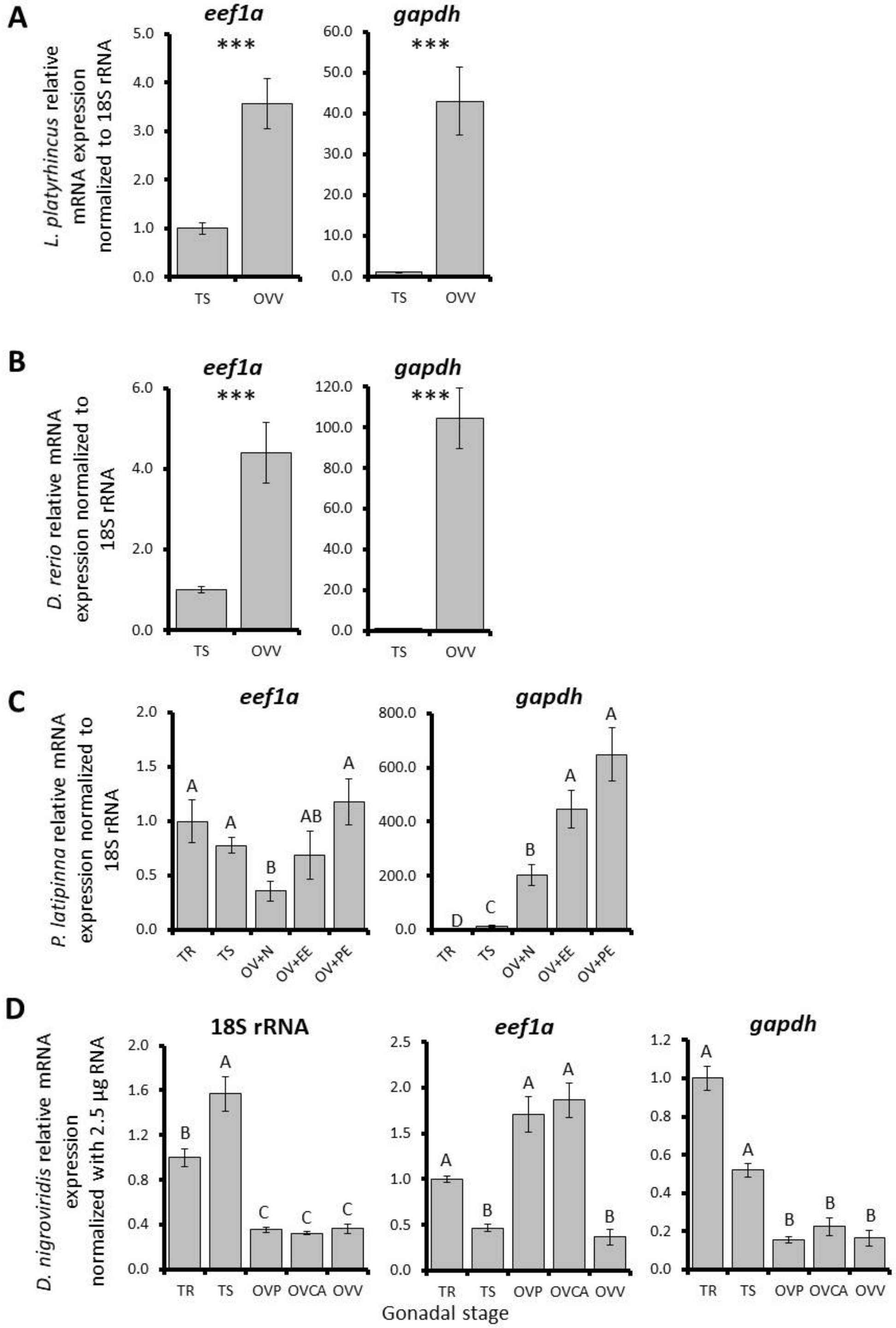

Fig. S2. Relative mRNA expression for putative qPCR reference genes that did not exhibit stability across organs in A) African turquoise killifish (*Nothobranchius furzeri*) and B) green-spotted puffer (*Dichotomyctere nigroviridis*). Each assay was normalized to input RNA quantity. Each bar represents the mean ± standard error, and different letters indicate significant differences ($p < 0.05$).

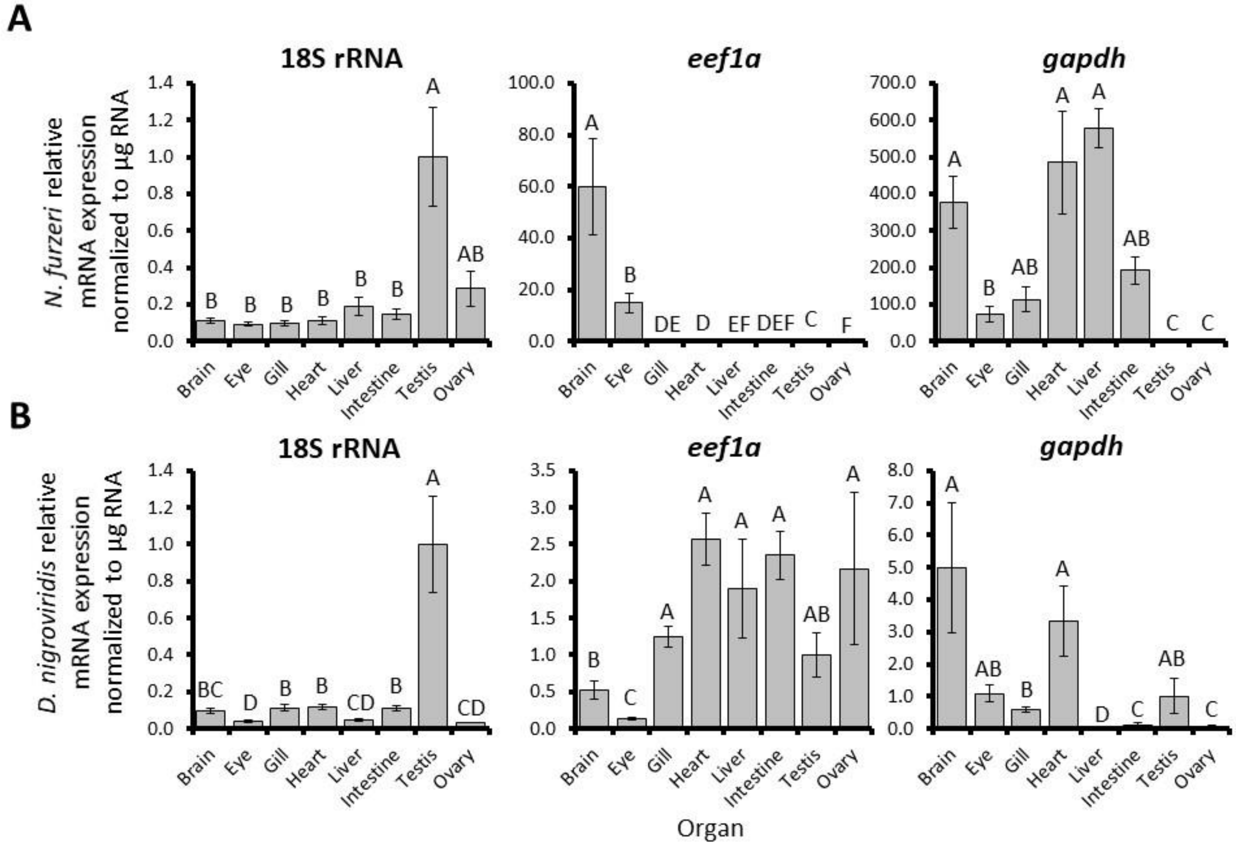

Fig. S3. Phylogenetic analysis of all verified fish *sreb* sequences in Ensembl version 99 (see Supplementary Dataset 1). The tree was generated using a maximum likelihood approach with 1000 bootstraps and rooted to hagfish (*Eptatretus burgeri*) *sreb*-like1.

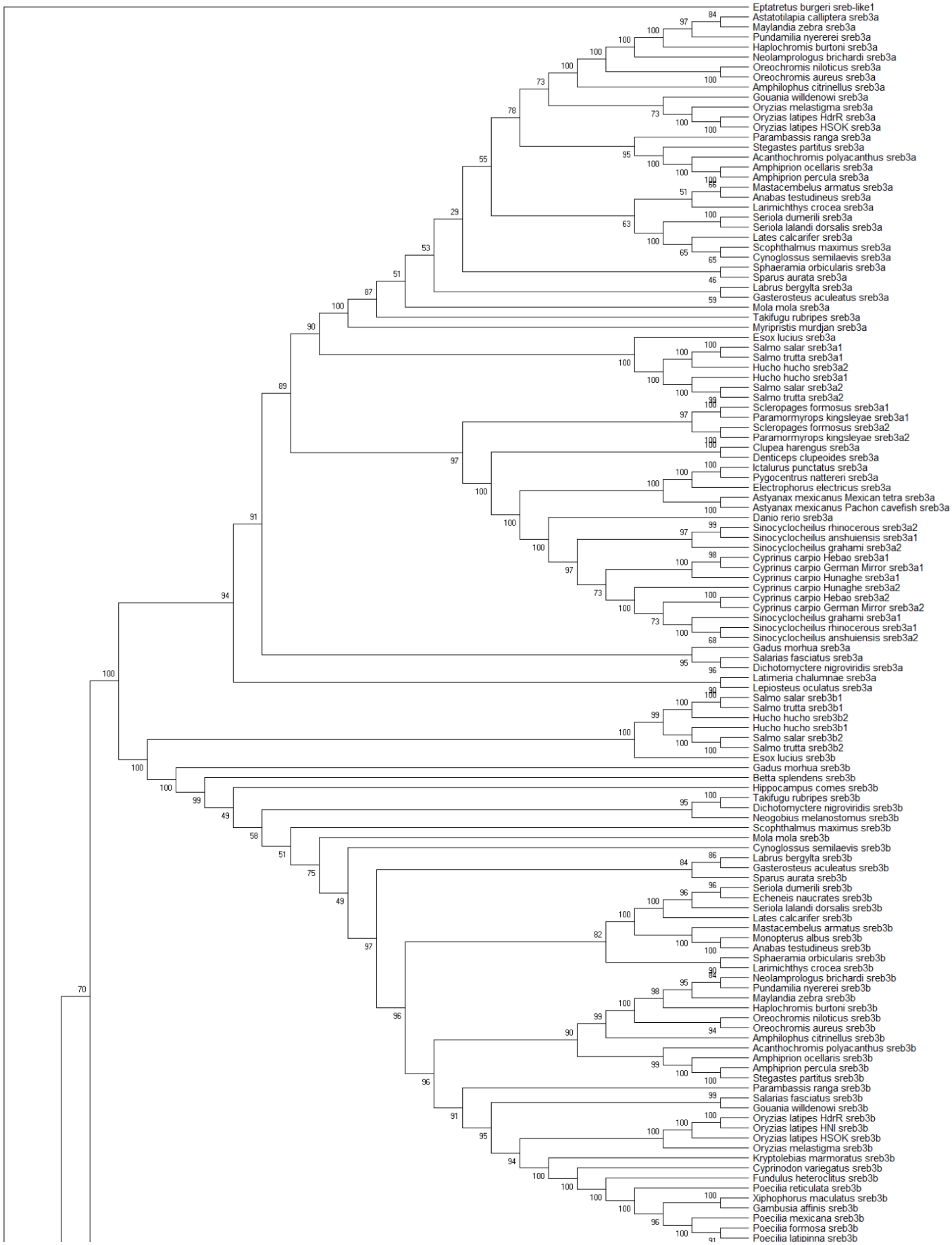

Fig. S3, cont.

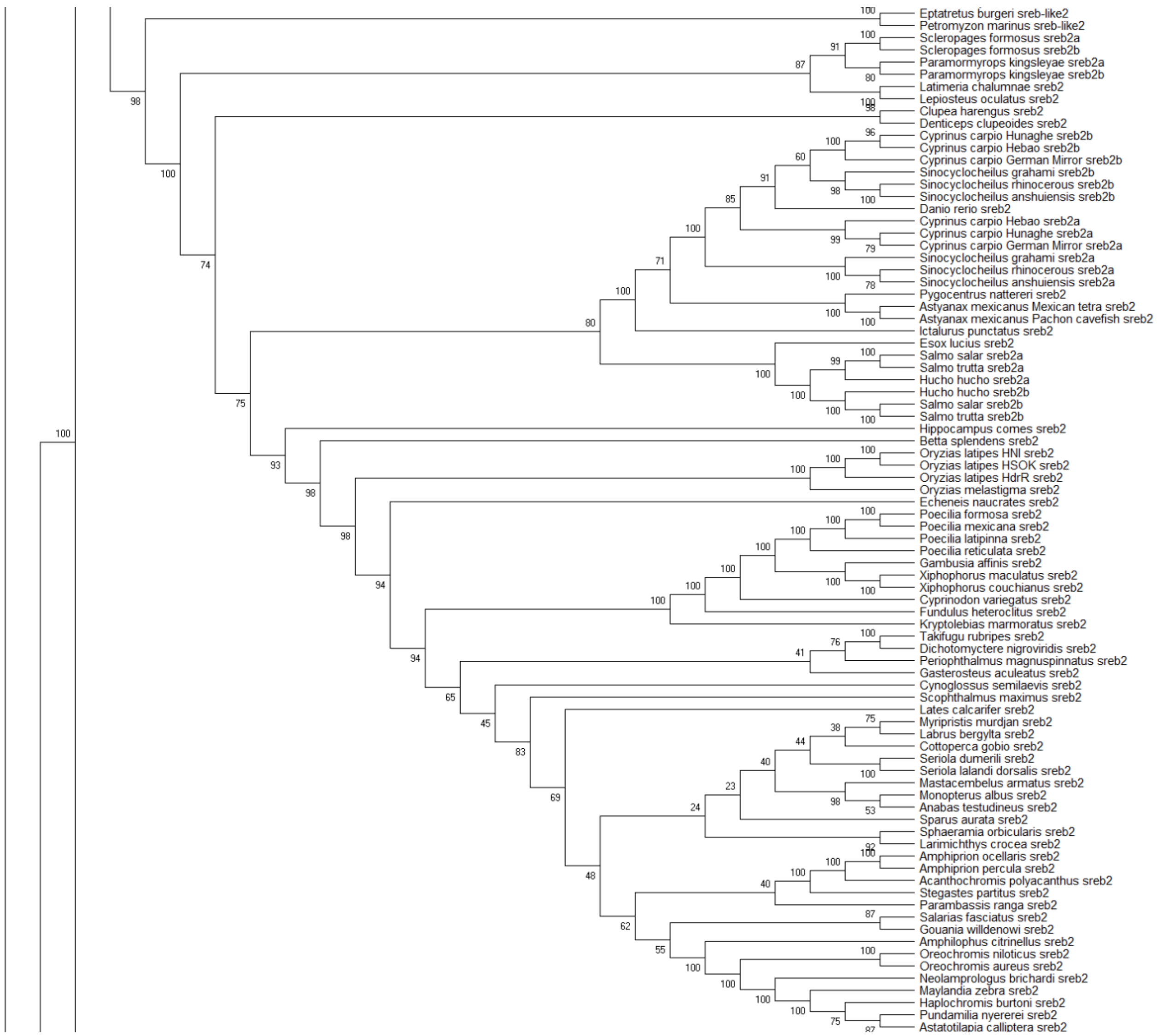
Eptatretus burgeri sreb-like2
Petromyzon marinus sreb-like2
Scleropages formosus sreb2a
Scleropages formosus sreb2b
Paramormyrops kingsleyae sreb2a
Paramormyrops kingsleyae sreb2b
Latimeria chalumnae sreb2
Lepiosteus oculatus sreb2
Clupea harengus sreb2
Denticeps clupeoides sreb2
Cyprinus carpio Hunaghe sreb2b
Cyprinus carpio Hebao sreb2b
Cyprinus carpio German Mirror sreb2b
Sinocyclocheilus grahami sreb2b
Sinocyclocheilus rhinocerous sreb2b
Sinocyclocheilus anshuiensis sreb2b
Danio rerio sreb2
Cyprinus carpio Hebao sreb2a
Cyprinus carpio Hunaghe sreb2a
Cyprinus carpio German Mirror sreb2a
Sinocyclocheilus grahami sreb2a
Sinocyclocheilus rhinocerous sreb2a
Sinocyclocheilus anshuiensis sreb2a
Pygocentrus nattereri sreb2
Astyanax mexicanus Mexican tetra sreb2
Astyanax mexicanus Pachon cavefish sreb2
Ictalurus punctatus sreb2
Esox lucius sreb2
Salmo salar sreb2a
Salmo trutta sreb2a
Hucho hucho sreb2a
Hucho hucho sreb2b
Salmo salar sreb2b
Salmo trutta sreb2b
Hippocampus comes sreb2
Betta splendens sreb2
Oryzias latipes HNI sreb2
Oryzias latipes HSOK sreb2
Oryzias latipes HdrR sreb2
Oryzias melastigma sreb2
Echeneis naucrates sreb2
Poecilia formosa sreb2
Poecilia mexicana sreb2
Poecilia latipinna sreb2
Poecilia reticulata sreb2
Gambusia affinis sreb2
Xiphophorus maculatus sreb2
Xiphophorus couchianus sreb2
Cyprinodon variegatus sreb2
Fundulus heteroclitus sreb2
Kryptolebias marmoratus sreb2
Takifugu rubripes sreb2
Dichotomyctere nigroviridis sreb2
Periophthalmus magnuspinnatus sreb2
Gasterosteus aculeatus sreb2
Cynoglossus semilaevis sreb2
Scophthalmus maximus sreb2
Lates calcarifer sreb2
Myripristis murdjan sreb2
Labrus bergylta sreb2
Cottoperca gobio sreb2
Seriola dumerili sreb2
Seriola lalandi dorsalis sreb2
Mastacembelus armatus sreb2
Monopterus albus sreb2
Anabas testudineus sreb2
Sparus aurata sreb2
Sphaeramia orbicularis sreb2
Larimichthys crocea sreb2
Amphiprion ocellaris sreb2
Amphiprion percula sreb2
Acanthochromis polyacanthus sreb2
Stegastes partitus sreb2
Parambassis ranga sreb2
Salarias fasciatus sreb2
Gouania willdenowi sreb2
Amphilophus citrinellus sreb2
Oreochromis niloticus sreb2
Oreochromis aureus sreb2
Neolamprologus brichardi sreb2
Maylandia zebra sreb2
Haplochromis burtoni sreb2
Pundamilia nyererei sreb2
Astatotilapia calliptera sreb2

Fig. S3, cont.

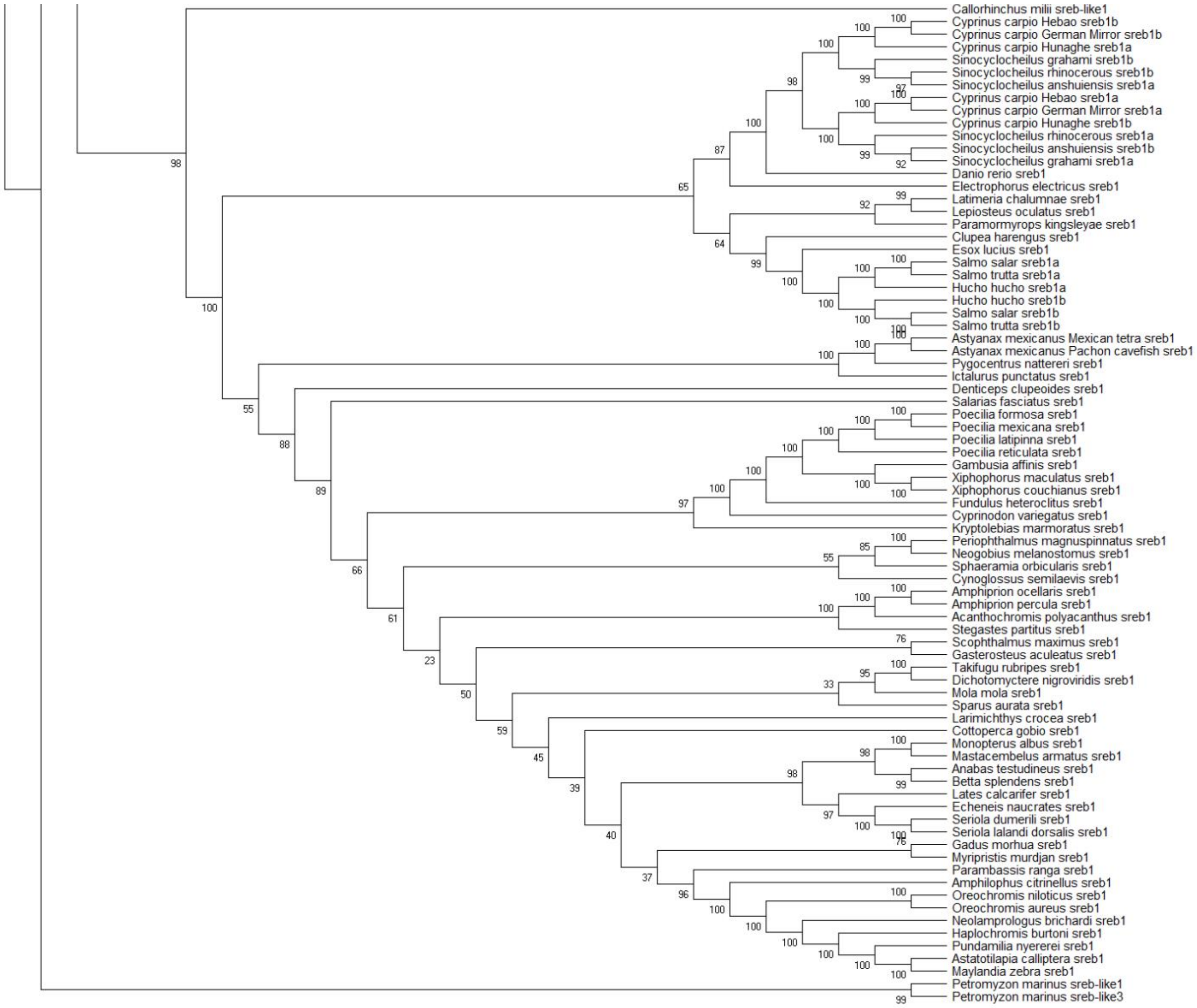

Callorhinchus milii sreb-like1
Cyprinus carpio Hebao sreb1b
Cyprinus carpio German Mirror sreb1b
Cyprinus carpio Hunaghe sreb1a
Sinocyclocheilus grahami sreb1b
Sinocyclocheilus rhinocerous sreb1b
Sinocyclocheilus anshuiensis sreb1a
Cyprinus carpio Hebao sreb1a
Cyprinus carpio German Mirror sreb1a
Cyprinus carpio Hunaghe sreb1b
Sinocyclocheilus rhinocerous sreb1a
Sinocyclocheilus anshuiensis sreb1b
Sinocyclocheilus grahami sreb1a
Danio rerio sreb1
Electrophorus electricus sreb1
Latimeria chalumnae sreb1
Lepiosteus oculatus sreb1
Paramormyrops kingsleyae sreb1
Clupea harengus sreb1
Esox lucius sreb1
Salmo salar sreb1a
Salmo trutta sreb1a
Hucho hucho sreb1a
Hucho hucho sreb1b
Salmo salar sreb1b
Salmo trutta sreb1b
Astyanax mexicanus Mexican tetra sreb1
Astyanax mexicanus Pachon cavefish sreb1
Pygocentrus nattereri sreb1
Ictalurus punctatus sreb1
Denticeps clupeoides sreb1
Salarias fasciatus sreb1
Poecilia formosa sreb1
Poecilia mexicana sreb1
Poecilia latipinna sreb1
Poecilia reticulata sreb1
Gambusia affinis sreb1
Xiphophorus maculatus sreb1
Xiphophorus couchianus sreb1
Fundulus heteroclitus sreb1
Cyprinodon variegatus sreb1
Kryptolebias marmoratus sreb1
Periophthalmus magnuspinnatus sreb1
Neogobius melanostomus sreb1
Sphaeramia orbicularis sreb1
Cynoglossus semilaevis sreb1
Amphiprion ocellaris sreb1
Amphiprion percula sreb1
Acanthochromis polyacanthus sreb1
Stegastes partitus sreb1
Scophthalmus maximus sreb1
Gasterosteus aculeatus sreb1
Takifugu rubripes sreb1
Dichotomyctere nigroviridis sreb1
Mola mola sreb1
Sparus aurata sreb1
Larimichthys crocea sreb1
Cottoperca gobio sreb1
Monopterus albus sreb1
Mastacembelus armatus sreb1
Anabas testudineus sreb1
Betta splendens sreb1
Lates calcarifer sreb1
Echeneis naucrates sreb1
Seriola dumerili sreb1
Seriola lalandi dorsalis sreb1
Gadus morhua sreb1
Myripristis murdjan sreb1
Parambassis ranga sreb1
Amphilophus citrinellus sreb1
Oreochromis niloticus sreb1
Oreochromis aureus sreb1
Neolamprologus brichardi sreb1
Haplochromis burtoni sreb1
Pundamilia nyererei sreb1
Astatotilapia calliptera sreb1
Maylandia zebra sreb1
Petromyzon marinus sreb-like1
Petromyzon marinus sreb-like3
